# Prognostic value of extracellular matrix gene mutations and expression in multiple myeloma

**DOI:** 10.1038/s41408-023-00817-7

**Published:** 2023-03-23

**Authors:** Marietheres Evers, Martin Schreder, Thorsten Stühmer, Franziska Jundt, Regina Ebert, Tanja Nicole Hartmann, Michael Altenbuchinger, Martina Rudelius, Martin Kuric, Wyonna Darleen Rindt, Torsten Steinbrunn, Christian Langer, Sofia Catalina Heredia-Guerrero, Hermann Einsele, Ralf Christian Bargou, Andreas Rosenwald, Ellen Leich

**Affiliations:** 1 Institute of Pathology, University of Würzburg, Würzburg, Germany; 2 Department of Internal Medicine II, University Hospital of Würzburg, Würzburg, Germany; 3 First Department of Medicine, Klinik Ottakring, Vienna, Austria; 4 Comprehensive Cancer Center Mainfranken, University Hospital of Würzburg, Würzburg, Germany; 5 Department of Musculoskeletal Tissue Regeneration, University of Würzburg, Würzburg, Germany; 6 Department of Internal Medicine I, Medical Center and Faculty of Medicine, University of Freiburg, Freiburg im Breisgau, Germany; 7 Department of Medical Bioinformatics, University Medical Center Göttingen, Göttingen, Germany; 8 Institute of Pathology, Ludwig-Maximilians-University München, München, Germany; 9 Department of Medical Oncology, Dana-Farber Cancer Institute, Harvard Medical School, Boston, MA USA; 10 Department of Internal Medicine III, University Hospital Ulm, Ulm, Germany

**Keywords:** Cancer genetics, Myeloma

Dear Editor,

Multiple Myeloma (MM) is characterized by a great clinical and genetic heterogeneity [1]. The disease remains largely uncurable, with drug resistance and patient relapse being major obstacles [2]. Therefore, it is important to uncover patient subgroups and targets for novel treatment approaches. In a previous whole exome sequencing (WES) study, an accumulation of single nucleotide variants (SNVs) in adhesion molecules was observed in MM [3, 4]. Since cell adhesion and the interaction of MM cells with the bone marrow microenvironment influence cell proliferation and survival as well as drug resistance [5–7], this study focused on the role of recurrently mutated and differentially expressed adhesion molecules to unveil potential prognostic markers or therapeutic targets in MM.

Mutations in adhesion genes were detected in 58 out of 67 samples from 43 patients of our previously published WES cohort [4] (Fig. 1A). Subsequent STRING network analysis for the mutated adhesion genes using high confidence settings revealed a clustering of SNVs in gene families associated with the extracellular matrix (ECM): Integrins, collagens, laminins, A disintegrin and metalloprotease (ADAM) as well as the ADAM with thrombospondin motifs (ADAMTS) family (Fig. 1B). Since the ECM gene families contain a total of 121 protein-coding genes covering a notable genomic area, we aimed to prove that there are more mutations within ECM genes than would be expected by chance. Thus, we determined the number of SNVs per protein length in all protein-coding genes sequenced within the Multiple Myeloma Research Foundation (MMRF) CoMMpass study (IA15 cohort; 1192 samples from 984 MM patients) and performed a Mann-Whitney-*U* test (Fig. 1C) which revealed significantly more SNVs per protein length in ECM genes compared to non-ECM genes (*p* = 0.000002), suggesting that ECM mutations might be potential cancer driver mutations.

**Fig. 1:**
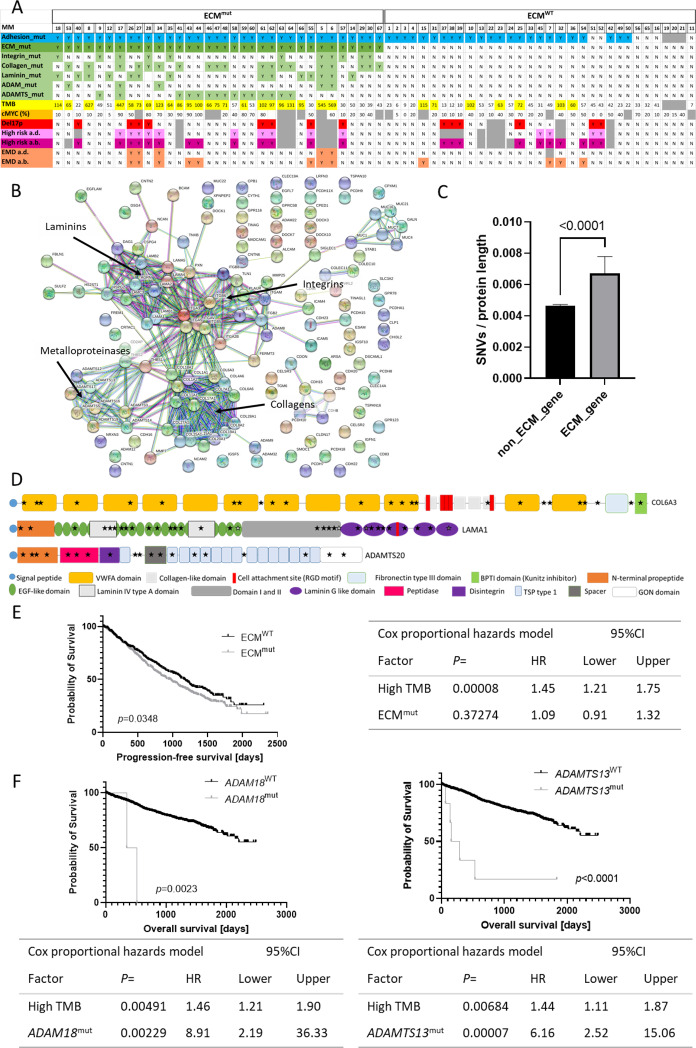
ECM gene mutations as prognostic markers in MM. **A** Representation of the mutation status of patients with and without mutations in adhesion genes (blue), ECM genes (green) (ECM^mut^ and ECM^WT^) and the TMB (yellow). Further, cMyc protein expression (orange), del17p status (red), overall high-risk status at diagnosis (a.d.) (light pink) and at biopsy (a.b.) (dark pink) and information on the presence of extramedullary disease (EMD) (salmon) are shown. Individual patients are separated by small gaps. **B** STRING network analysis of mutated adhesion genes in our WES study cohort revealed clusters in integrin, collagen, laminin and metalloproteinase genes (ADAMs, ADAMTS). **C** ECM genes contained significantly more SNVs relative to the protein length than other protein-coding genes within the MMRF WES dataset. Non-protein-coding genes were excluded from the analysis (Table S1). Data shown is median with 95% CI. Statistical test was Mann-Whitney-*U*. **D** Visual representation of domains and structure of proteins encoded by the three ECM genes most recurrently affected by mutations: *COL6A3*, *LAMA1* and *ADAMTS20*. For all other ECM molecules and more information on mutations see Fig. S1 and Table S3. Information on protein structure and domains was obtained from the Uniprot knowledgebase. **E** PFS was significantly shorter (median PFS 938.0 vs. 1176.0 days) in ECM^mut^ patients compared to ECM^WT^ patients. Comparisons were performed using the Kaplan Meier method and Log Rank test. Table shows Hazard ratios (HR) for high TMB and ECM mutations calculated using the Cox proportional hazards model in SPSS. **F** Mutations in *ADAM18* and *ADAMTS13* were associated with a significantly shorter OS (median 349.0 days vs. not reached and 154.0 vs. not reached, respectively). Tables below Kaplan Meier plots show HRs calculated using the Cox proportional hazards model for high TMB and *ADAM18*^mut^ or *ADAMTS13*^mut^. Graphs were created using GraphPad Prism 9.

Subsequently, mutation frequencies and the distribution within genes of the ECM gene families were assessed in our dataset and the MMRF dataset. Mutations were detected at similar frequencies in both cohorts: 58.1% of patients from our cohort had a mutation in at least one of the ECM gene families (ECM^mut^) (56.5% in the MMRF cohort). Collagens were the most frequently mutated gene family (30.2% of patients in our dataset and 30.4% in the MMRF cohort), followed by laminins (25.6% and 12.9%), ADAMTS (20.9% and 16.1%), integrins (20.9% and 13.3%) and ADAMs (9.3% and 7.5%) (Table S2).

The most recurrently mutated gene, *LAMA1*, was mutated in 2.95% of patients within the MMRF cohort (Table S2). *COL6A3*, *COL6A5*, *COL12A1*, *LAMA1*, *ADAMTS2* and *ADAMTS20* were mutated in more than 2% of patients (Table S2). Generally, the mutations were widely spread across the different ECM genes and no classical hotspots or clustering of mutations in particular protein domains were observed within the individual genes (Fig. 1D, Table S3, Fig. S1). However, mutations were detected in the same codon in more than one patient in eight ECM genes, translating to the p.R977Q variant in ITGA2B, p.A757V/T in ITGB2, p.L246V/R in LAMB3, p.R215H in COL22A1, p.R504Q in ADAM11, p.V387I in ADAMTS1, p.A1005T/V in ADAMTS2 and p.R232H in ADAMTS7 (Table S3).

To determine factors distinguishing ECM^mut^ from ECM^WT^ patients, the ECM mutation status was correlated with molecular parameters (Fig. 1A, Table S4). We found that ECM^mut^ samples had a significantly higher tumor mutational burden (TMB, defined as the sum of all SNVs detected in one sample) compared to ECM^WT^ samples in both our dataset (two-sided Fisher’s exact test; Median 70.0 vs. 42.5; *p* = 0.000178, *n* = 64) and in the MMRF cohort (median 65.0 vs. 51.5; *p* < 0.0001, *n* = 1192). In line with previous findings [8], MMRF patients with a high TMB (high defined as higher than the median in at least one sample) had a significantly shorter overall survival (OS) (median 2150 days vs not reached; *p* = 0.003) and progression-free survival (PFS) (median 883 vs 1228 days; *p* < 0.0001) compared to patients with a consistently low TMB.

Other high-risk features such as high cMyc expression, the del17p alone or in combination with TP53-mutation (double-hit), the occurrence of extramedullary disease or other classical cytogenetic parameters were not significantly enriched in ECM^mut^ samples in our dataset after Benjamini-Hochberg (BH) correction for multiple hypotheses (Table S4).

Subsequently, the MMRF cohort was used to correlate mutation status with PFS and OS. PFS was significantly shorter in patients with ECM gene mutations, while OS was not significantly affected (Fig. 1E, Table S2). Further analysis revealed no significant association between mutations in the individual ECM gene families (Collagens, ADAMs, etc.) and survival after correction for multiple hypotheses (Table S2). Multivariate analysis using the Cox regression method only corroborated high TMB as a significantly bad prognostic marker (Fig. 1E).

Focusing on individual genes, we found that patients with mutations in *ADAM18* and *ADAMTS13* had a significantly shorter OS after correction for multiple hypotheses (Fig. 1F, Table S2). Moreover, Cox regression identified *ADAM18* and *ADAMTS13* mutations as bad prognostic factors, independent from TMB (Fig. 1F).

Having analyzed the role of ECM gene mutations, we subsequently assessed ECM gene expression (GE) using the MMRF RNA-sequencing dataset (estimates calculated using Salmon 0.7.2).

Considerable levels of GE were observed for many integrins, with *ITGA3*, *ITGA4*, *ITGA6*, *ITGA8*, *ITGAE*, *ITGAL*, *ITGAV*, *ITGB1*, *ITGB2* and *ITGB7* being the most highly expressed integrins (median 3.76–46.75 transcripts per kilobase million (TPM)) (Fig. 2A, Table S5). Most laminin genes were expressed at very low levels (median < 1 TPM) except for *LAMA5* and *LAMC1* (Fig. 2B). ADAM genes with a notable expression (median > 3 TPM) included *ADAM8*, *ADAM9*, *ADAM10*, *ADAM15*, *ADAM17*, *ADAM19* and *ADAM28*, with *ADAM10* being the most highly expressed gene (median 20.35 TPM) (Fig. 2C). Within the ADAMTS and collagen families, only *ADAMTS13*, *COL4A3*, *COL4A4*, *COL4A5*, *COL7A1*, *COL9A2*, *COL9A3* and *COL24A1* were expressed at considerable levels (median > 1 TPM) (Fig. 2D, E). GE data is summarized in Table S5.

**Fig. 2:**
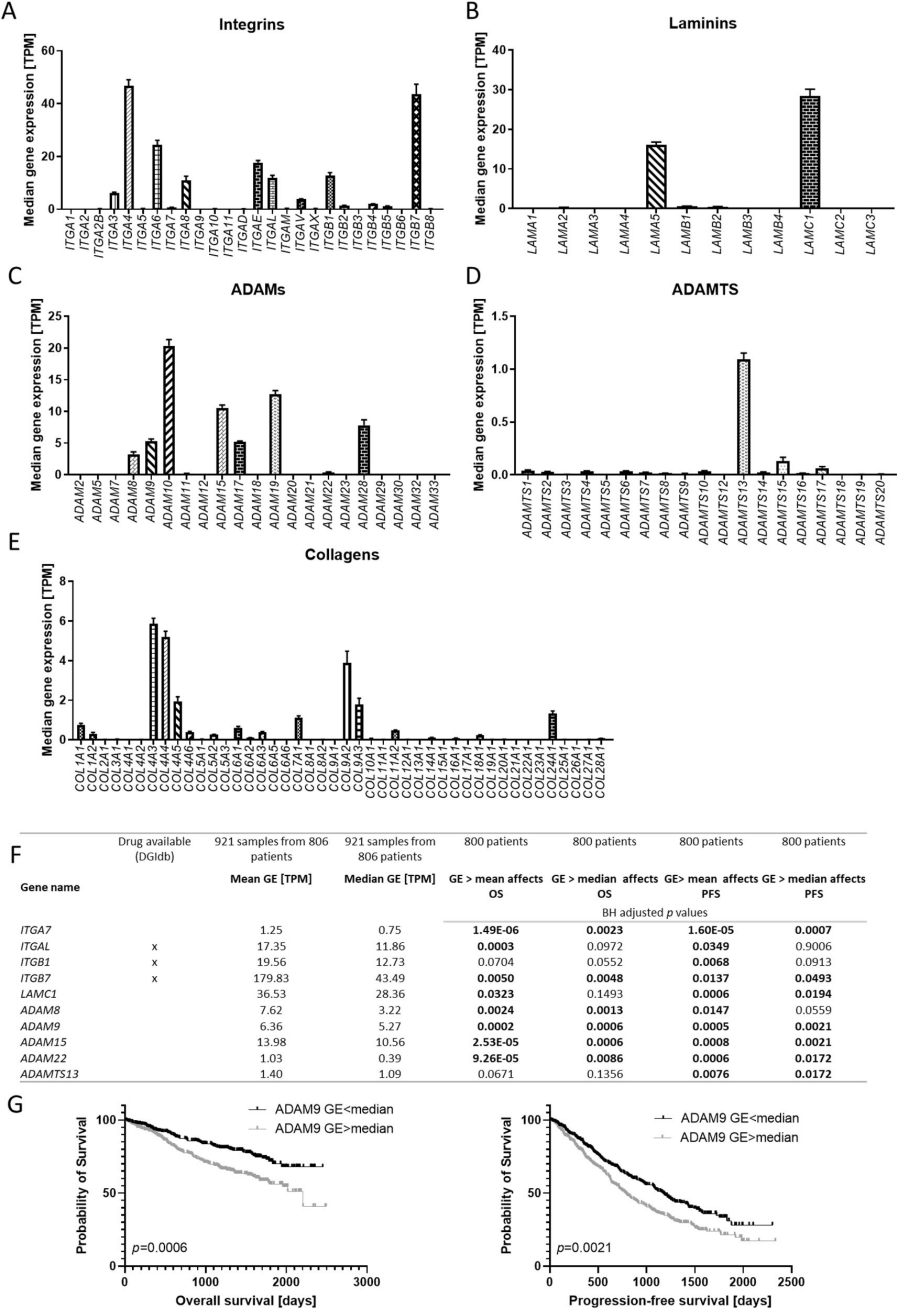
ECM gene expression levels as prognostic markers in MM. Gene expression of integrin (**A**), laminin (**B**), ADAM (**C**), ADAMTS (**D**) and collagen (**E**) genes determined by RNA sequencing of 921 samples from 806 patients of the MMRF cohort. Data shown is median transcripts per kilobase million (TPM) with 95% CI. **F** Summary of selected genes where a GE higher than the mean or median is significantly associated with PFS and/or OS. Comparisons were performed using the Kaplan Meier method and Log Rank test. *P* values were adjusted for multiple hypotheses using the Benjamini-Hochberg (BH) correction in R. For Kaplan Meier plots see Fig. S2. DGIdb was used to reveal druggable targets. **G** Exemplary Kaplan Meier plots comparing PFS and OS of patients with a high (>median) or low (<median) *ADAM9* GE. Statistical test was Log Rank test. Graphs were created using GraphPad Prism 9.

The effect of a high or low GE of each single ECM gene (> or < median or mean GE measured across all samples) on PFS and OS was assessed for all patients where both GE and survival data was available (*n* = 800 patients). The median GE of all longitudinal samples was used for patients with more than one sample available. High GE ( > mean and/or median) of 9/26 integrins, 15/44 collagens, 7/12 laminins, 8/20 ADAMs and 7/19 ADAMTS genes significantly correlated with OS after correction for multiple hypotheses. PFS was also significantly shorter in patients with a high GE of 10/26 integrins, 11/44 collagens, 4/12 laminins, 7/20 ADAMs and 6/19 ADAMTS genes. *P* values are summarized in Table S5.

In order to identify the most promising potential therapeutic targets, we focused on genes with a considerable GE (median/mean TPM > 1), where an expression higher than the median or mean was associated with a significantly shorter PFS or OS (Table S5). To validate the finding that high GE of these genes was associated with worse survival, we increased GE thresholds for separating “high” from “low” GE systematically within the range of expression (e.g. TPM > 5, 20, 30 for *ITGAL*) and correlated the corresponding categorical variable with PFS and OS (Table S5). Notably, the most consistent correlations of high GE with survival were observed for *ITGA7*, *ITGAL*, *ITGB1*, *ITGB7*, *LAMC1*, *ADAM8*, *ADAM9*, *ADAM15*, *ADAM22*, *ADAMTS13* (Figs. 2F, G, S2, Table S5).

To our knowledge, this is the first study identifying ECM mutations as possible driver mutations. In line with that, high expression of a considerable amount of integrin and ADAM genes was associated with poor survival outcomes, suggesting that these genes may prove to be valuable prognostic markers as well as potential novel treatment targets that should be the focus of further functional research.

This is especially relevant for ADAMs, which are important for the development and progression of many cancer types [9], but whose role in MM is scarcely studied.

Supporting the reliability of the results described herein, a correlation of high *ITGB1* and ITGB7 expression with poor survival outcomes has already been described in other studies [10, 11]. ITGB7 has also been shown to play a role in MM cell adhesion, migration and invasion [12] and its activated conformation has been proposed as a target for CAR T cell therapy in MM [13]. Inhibition of integrin αLβ2 (αL encoded by *ITGAL*), whose expression is correlated with tumor growth, induces apoptosis in MM cells [14, 15].

In conclusion, this study shows that ECM genes are recurrently mutated and that the expression levels of several ECM molecules might be relevant prognostic markers or therapeutic targets in MM.

## Supplementary information

Supplementary Figures S1 and S2

Table S1

Table S2

Table S3

Table S4

Table S5

## Data Availability

Sequencing data for our dataset are available in the European Genome-phenome Archive (EGA) under accession number EGAS00001003227. The MMRF datasets analyzed during the current study are available at https://research.themmrf.org and www.themmrf.org.
