# Supplementary material for: Prognostic value of extracellular matrix gene mutations and expression in multiple myeloma
Source: Blood Cancer J. 2023 Mar 23;13(1):43. doi: 10.1038/s41408-023-00817-7 (PMC10036560; doi:10.1038/s41408-023-00817-7)
Supplement: Supplementary file 1 — Supplementary Figures S1 and S2 [file 41408_2023_817_MOESM1_ESM.pdf]

Figure S1: Visual representation of protein domains and structure of all ECM molecules. Information on protein domains was obtained from the UniProt knowledgebase (https://www.uniprot.org/). Locations of patient mutations found in the MMRF (black) and our dataset (orange) are marked with stars. Mutations within the same protein detected in the same patient are marked with grey stars. Images were created using PowerPoint.

Integrins

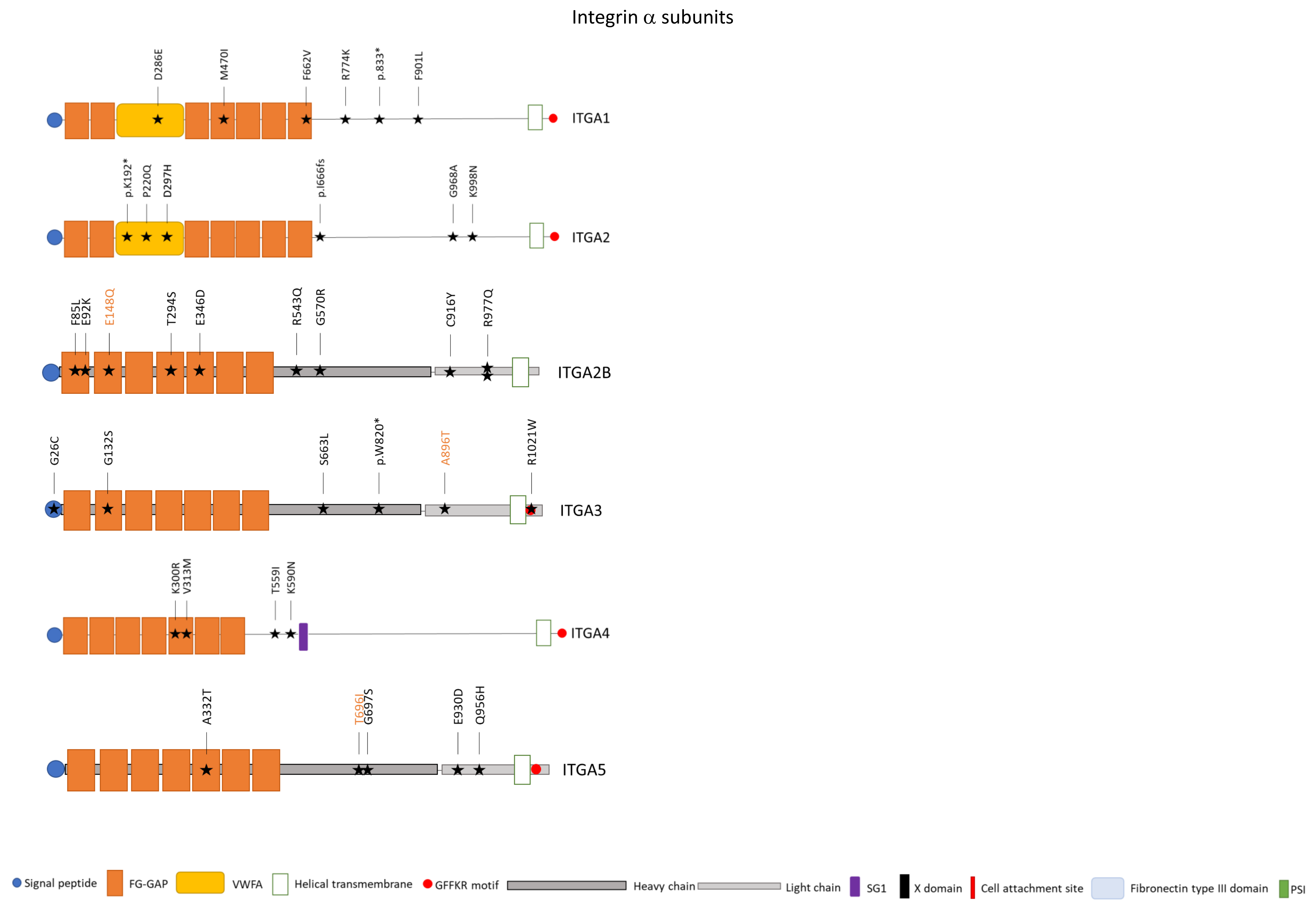

Integrin α subunits
D286E
M470I
F662V
R774K
p.833*
F901L
ITGA1
p.K192*
P220Q
D297H
p.I666fs
G968A
K998N
ITGA2
F85L
E92K
E148Q
T294S
E346D
R543Q
G570R
C916Y
R977Q
ITGA2B
G26C
G132S
S663L
p.W820*
A896T
R1021W
ITGA3
K300R
V313M
T559I
K590N
ITGA4
A332T
T696I
G697S
E930D
Q956H
ITGA5
Signal peptide
FG-GAP
VWFA
Helical transmembrane
GFFKR motif
Heavy chain
Light chain
SG1
X domain
Cell attachment site
Fibronectin type III domain
PSI

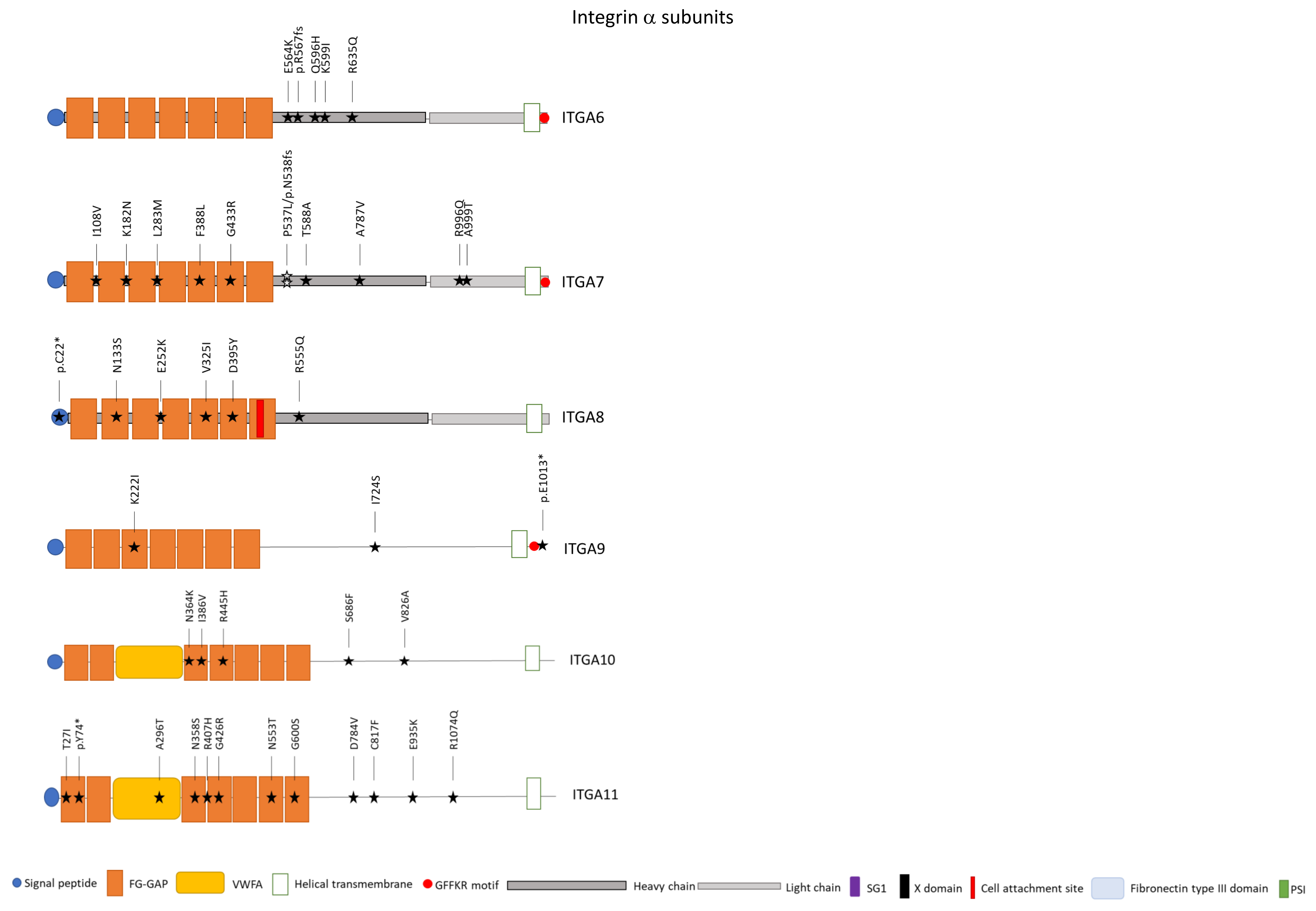
Integrin α subunits
E564K
p.R567fs
Q596H
K599I
R635Q
ITGA6
I108V
K182N
L283M
F388L
G433R
P537L/p.N538fs
T588A
A787V
R996Q
A999T
ITGA7
p.C22*
N133S
E252K
V325I
D395Y
R555Q
ITGA8
K222I
I724S
p.E1013*
ITGA9
N364K
I386V
R445H
S686F
V826A
ITGA10
T27I
p.Y74*
A296T
N358S
R407H
G426R
N553T
G600S
D784V
C817F
E935K
R1074Q
ITGA11
Signal peptide
FG-GAP
VWFA
Helical transmembrane
GFFKR motif
Heavy chain
Light chain
SG1
X domain
Cell attachment site
Fibronectin type III domain
PSI

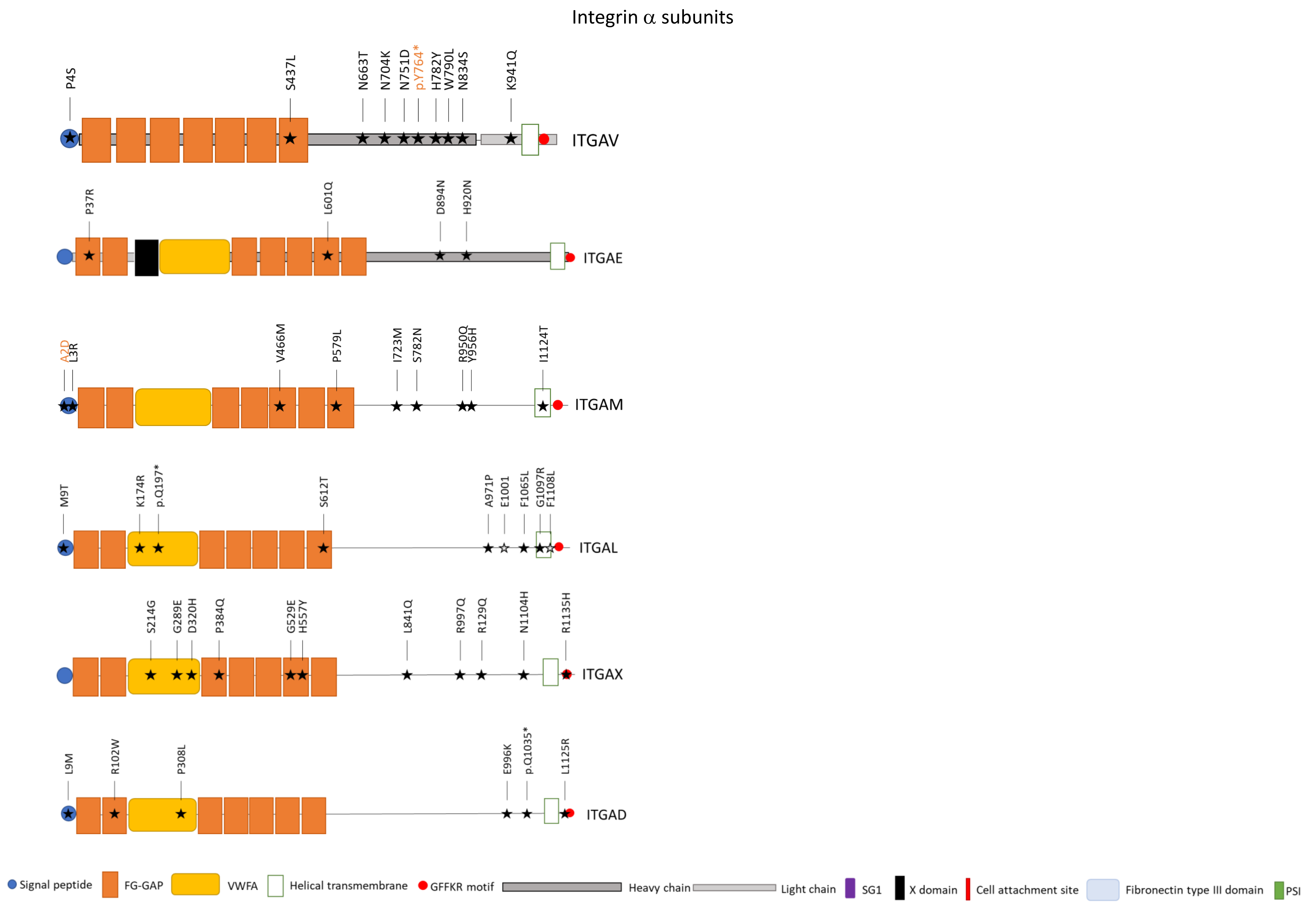

Integrin α subunits
P4S
S437L
N663T
N704K
N751D
p.Y764*
H782Y
W790L
N834S
K941Q
ITGAV
P37R
L601Q
D894N
H920N
ITGAE
A2D
L3R
V466M
P579L
I723M
S782N
R950Q
Y956H
I1124T
ITGAM
M9T
K174R
p.Q197*
S612T
A971P
E1001
F1065L
G1097R
F1108L
ITGAL
S214G
G289E
D320H
P384Q
G529E
H557Y
L841Q
R997Q
R129Q
N1104H
R1135H
ITGAX
L9M
R102W
P308L
E996K
p.Q1035*
L1125R
ITGAD
Signal peptide
FG-GAP
VWFA
Helical transmembrane
GFFKR motif
Heavy chain
Light chain
SG1
X domain
Cell attachment site
Fibronectin type III domain
PSI

Integrin β subunits

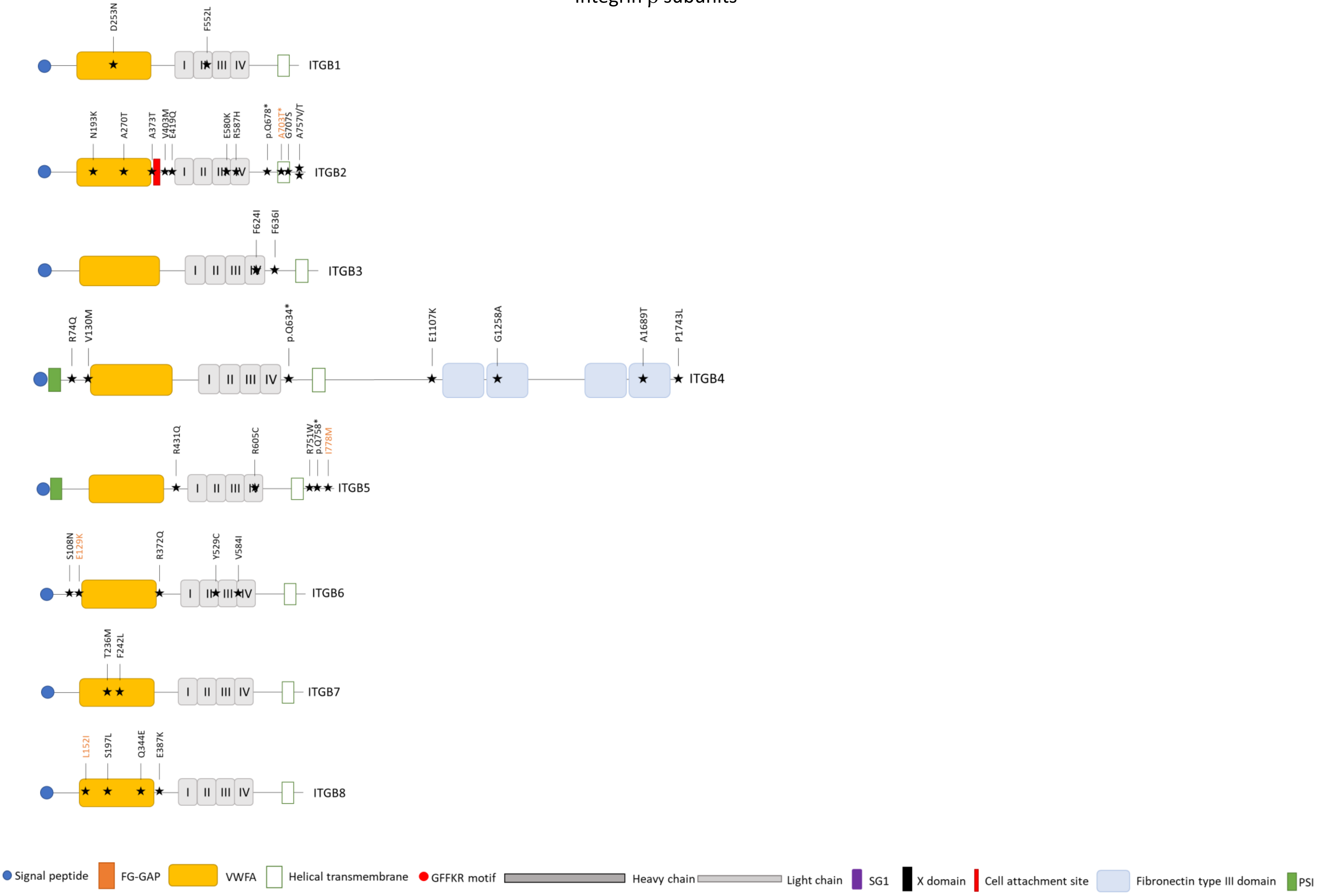

Collagens

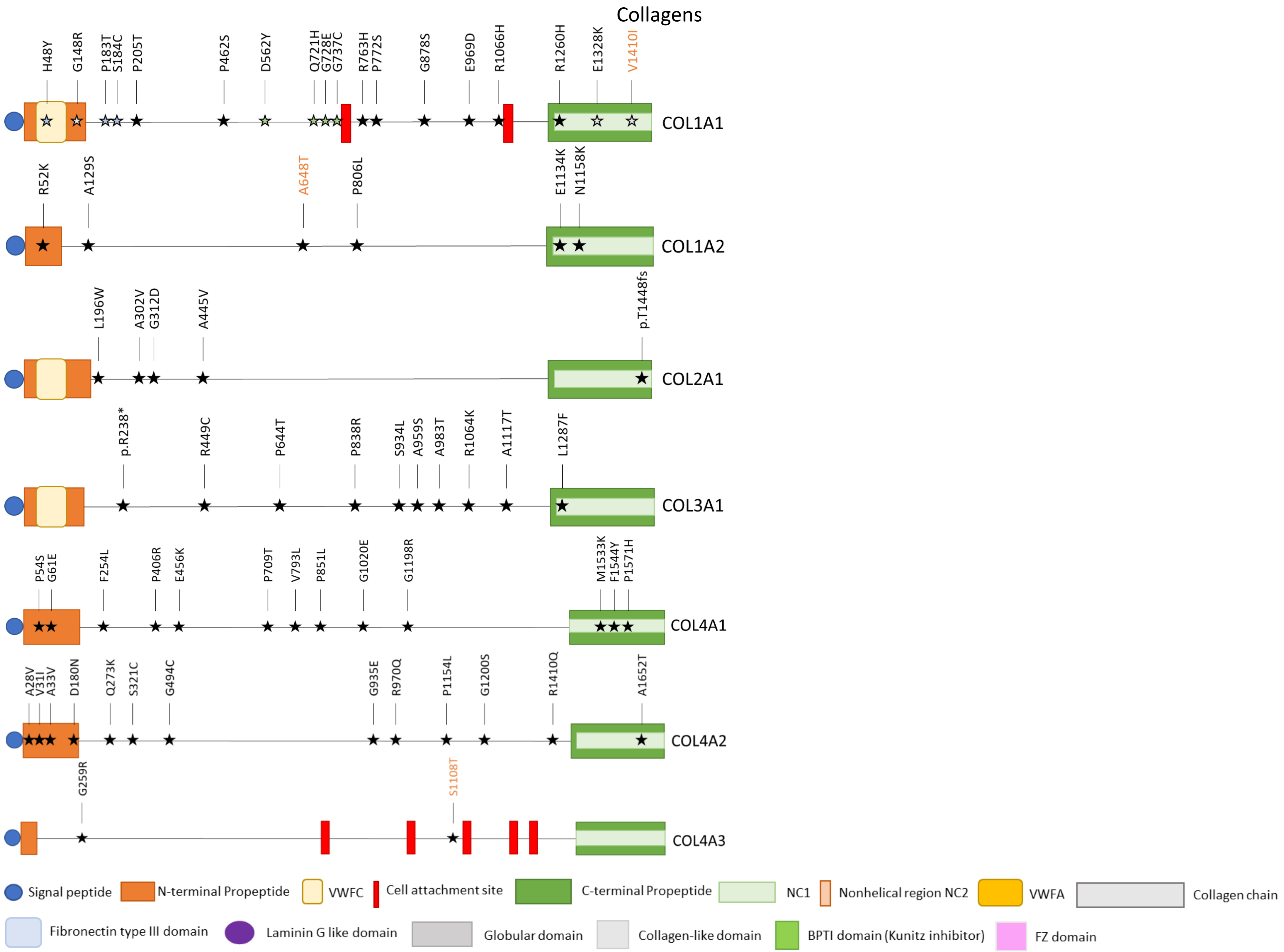
Collagens
H48Y
G148R
P183T
S184C
P205T
P462S
D562Y
Q721H
G728E
G737C
R763H
P772S
G878S
E969D
R1066H
R1260H
E1328K
V1410I
COL1A1
R52K
A129S
A648T
P806L
E1134K
N1158K
COL1A2
L196W
A302V
G312D
A445V
p.T1448fs
COL2A1
p.R238*
R449C
P644T
P838R
S934L
A959S
A983T
R1064K
A1117T
L1287F
COL3A1
P54S
G61E
F254L
P406R
E456K
P709T
V793L
P851L
G1020E
G1198R
M1533K
F1544Y
P1571H
COL4A1
A28V
V31I
A33V
D180N
Q273K
S321C
G494C
G935E
R970Q
P1154L
G1200S
R1410Q
A1652T
COL4A2
G259R
S1108T
COL4A3
Signal peptide
N-terminal Propeptide
VWFC
Cell attachment site
C-terminal Propeptide
NC1
Nonhelical region NC2
VWFA
Collagen chain
Fibronectin type III domain
Laminin G like domain
Globular domain
Collagen-like domain
BPTI domain (Kunitz inhibitor)
FZ domain

Collagens

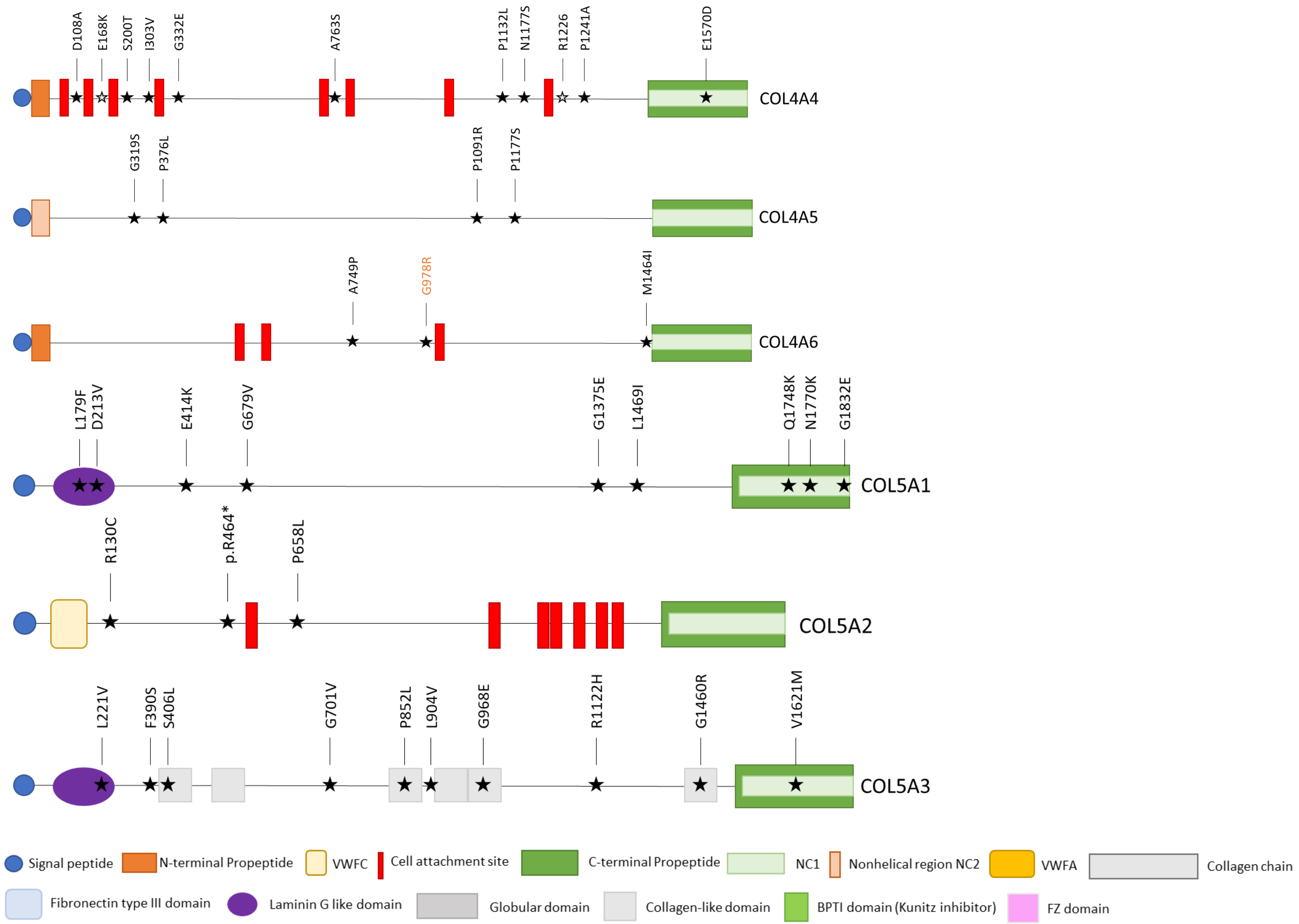
D108A
E168K
S200T
I303V
G332E
A763S
P1132L
N1177S
R1226
P1241A
E1570D
COL4A4
G319S
P376L
P1091R
P1177S
COL4A5
A749P
G978R
M1464I
COL4A6
L179F
D213V
E414K
G679V
G1375E
L1469I
Q1748K
N1770K
G1832E
COL5A1
R130C
p.R464*
P658L
COL5A2
L221V
F390S
S406L
G701V
P852L
L904V
G968E
R1122H
G1460R
V1621M
COL5A3
Signal peptide
N-terminal Propeptide
VWFC
Cell attachment site
C-terminal Propeptide
NC1
Nonhelical region NC2
VWFA
Collagen chain
Fibronectin type III domain
Laminin G like domain
Globular domain
Collagen-like domain
BPTI domain (Kunitz inhibitor)
FZ domain

Collagens

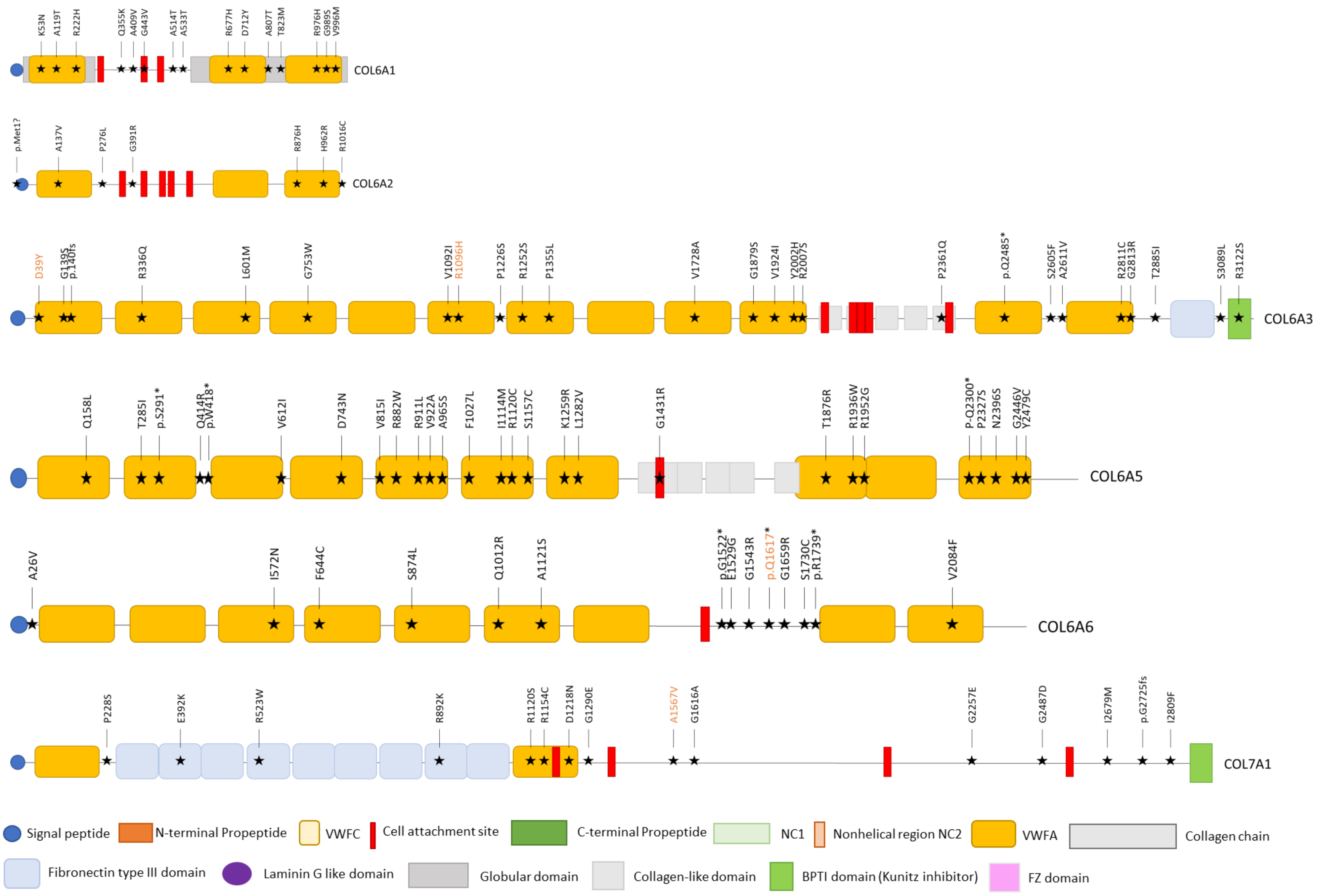
COL6A1
K53N
A119T
R222H
Q355K
A409V
G443V
A514T
A533T
R677H
D712Y
A807T
T823M
R976H
G989S
V996M
COL6A2
p.Met1?
A137V
P276L
G391R
R876H
H962R
R1016C
COL6A3
D39Y
G139S
p.140fs
R336Q
L601M
G753W
V1092I
R1096H
P1226S
R1252S
P1355L
V1728A
G1879S
V1924I
Y2002H
R2007S
P2361Q
p.Q2485*
S2605F
A2611V
R2811C
G2813R
T2885I
S3089L
R3122S
COL6A5
Q158L
T285I
p.S291*
Q414R
p.W418*
V612I
D743N
V815I
R882W
R911L
V922A
A965S
F1027L
I1114M
R1120C
S1157C
K1259R
L1282V
G1431R
T1876R
R1936W
R1952G
P-Q2300*
P2327S
N2396S
G2446V
Y2479C
COL6A6
A26V
I572N
F644C
S874L
Q1012R
A1121S
p.G1522*
E1529G
G1543R
p.Q1617*
G1659R
S1730C
p.R1739*
V2084F
COL7A1
P228S
E392K
R523W
R892K
R1120S
R1154C
D1218N
G1290E
A1567V
G1616A
G2257E
G2487D
I2679M
p.G2725fs
I2809F
Signal peptide
N-terminal Propeptide
VWFC
Cell attachment site
C-terminal Propeptide
NC1
Nonhelical region NC2
VWFA
Collagen chain
Fibronectin type III domain
Laminin G like domain
Globular domain
Collagen-like domain
BPTI domain (Kunitz inhibitor)
FZ domain

Collagens

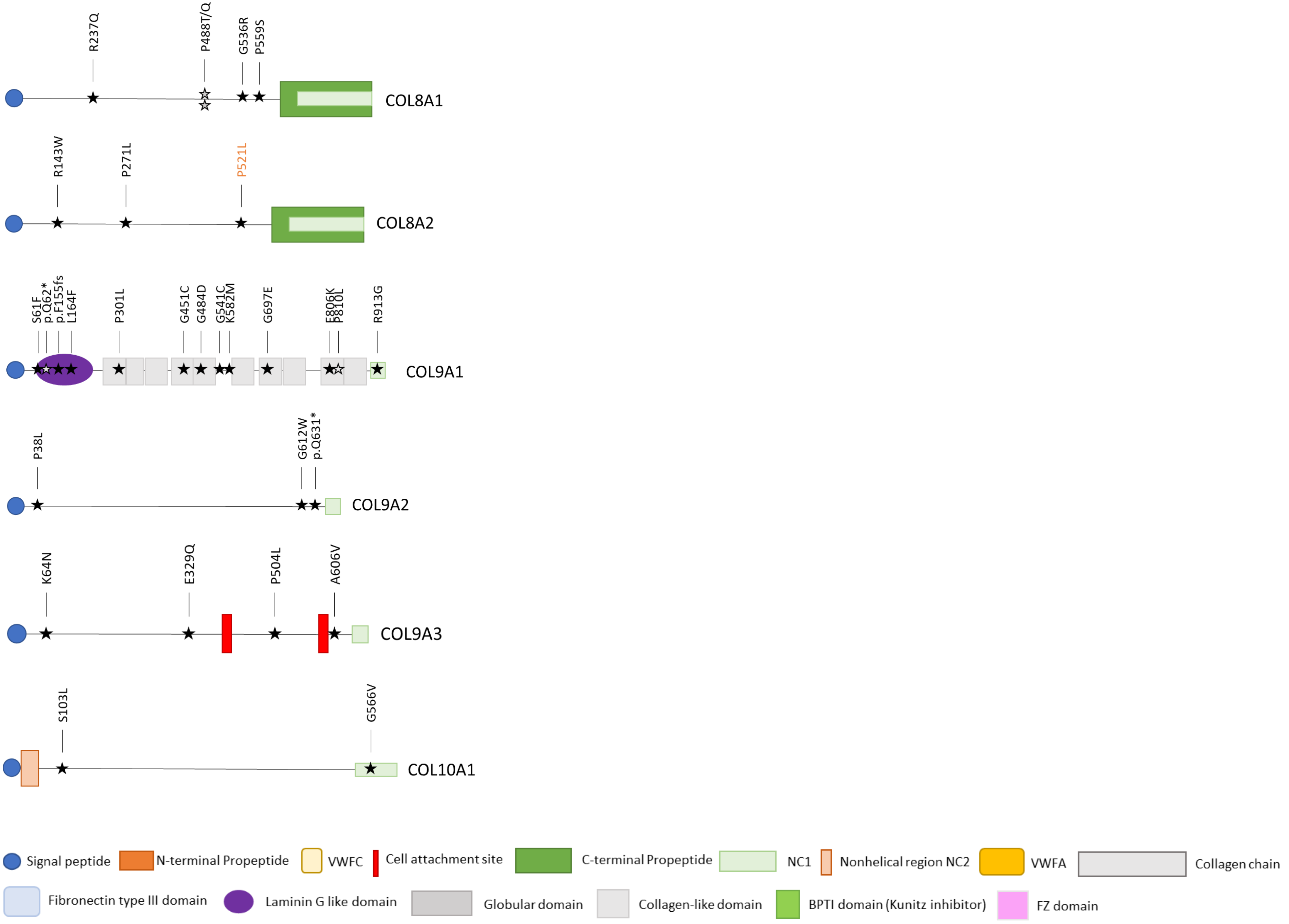
R237Q
P488T/Q
G536R
P559S
COL8A1
R143W
P271L
P521L
COL8A2
S61F
p.Q62*
p.F155fs
L164F
P301L
G451C
G484D
G541C
K582M
G697E
E806K
P810L
R913G
COL9A1
P38L
G612W
p.Q631*
COL9A2
K64N
E329Q
P504L
A606V
COL9A3
S103L
G566V
COL10A1
Signal peptide
N-terminal Propeptide
VWFC
Cell attachment site
C-terminal Propeptide
NC1
Nonhelical region NC2
VWFA
Collagen chain
Fibronectin type III domain
Laminin G like domain
Globular domain
Collagen-like domain
BPTI domain (Kunitz inhibitor)
FZ domain

Collagens

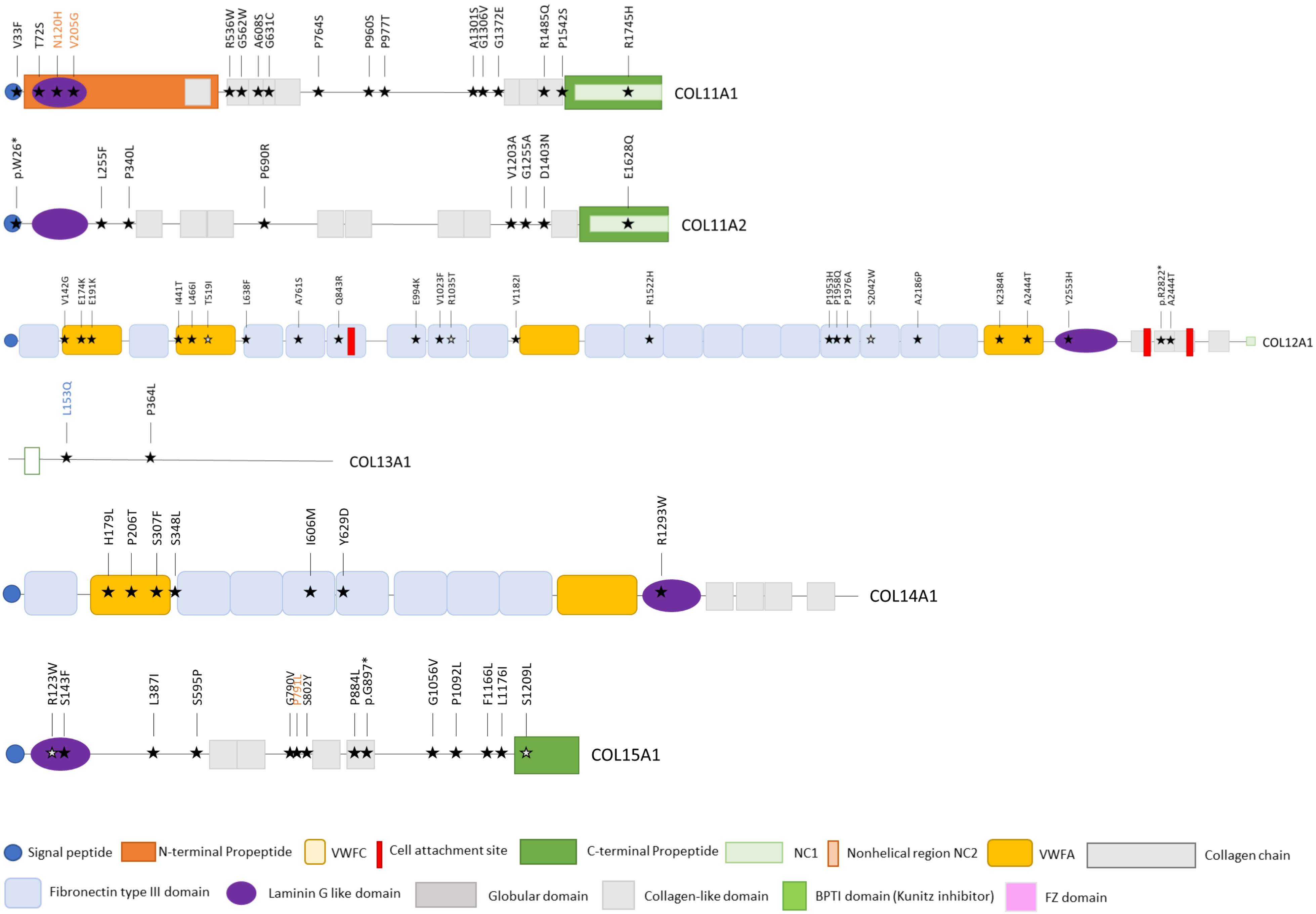

V33F
T72S
N120H
V205G
R536W
G562W
A608S
G631C
P764S
P960S
P977T
A1301S
G1306V
G1372E
R1485Q
P1542S
R1745H
COL11A1
p.W26*
L255F
P340L
P690R
V1203A
G1255A
D1403N
E1628Q
COL11A2
V142G
E174K
E191K
I441T
L466I
T519I
L638F
A761S
Q843R
E994K
V1023F
R1035T
V1182I
R1522H
P1953H
P1958Q
P1976A
S2042W
A2186P
K2384R
A2444T
Y2553H
p.R2822*
A2444T
COL12A1
L153Q
P364L
COL13A1
H179L
P206T
S307F
S348L
I606M
Y629D
R1293W
COL14A1
R123W
S143F
L387I
S595P
G790V
P791L
S802Y
P884L
p.G897*
G1056V
P1092L
F1166L
L1176I
S1209L
COL15A1
Signal peptide
N-terminal Propeptide
VWFC
Cell attachment site
C-terminal Propeptide
NC1
Nonhelical region NC2
VWFA
Collagen chain
Fibronectin type III domain
Laminin G like domain
Globular domain
Collagen-like domain
BPTI domain (Kunitz inhibitor)
FZ domain

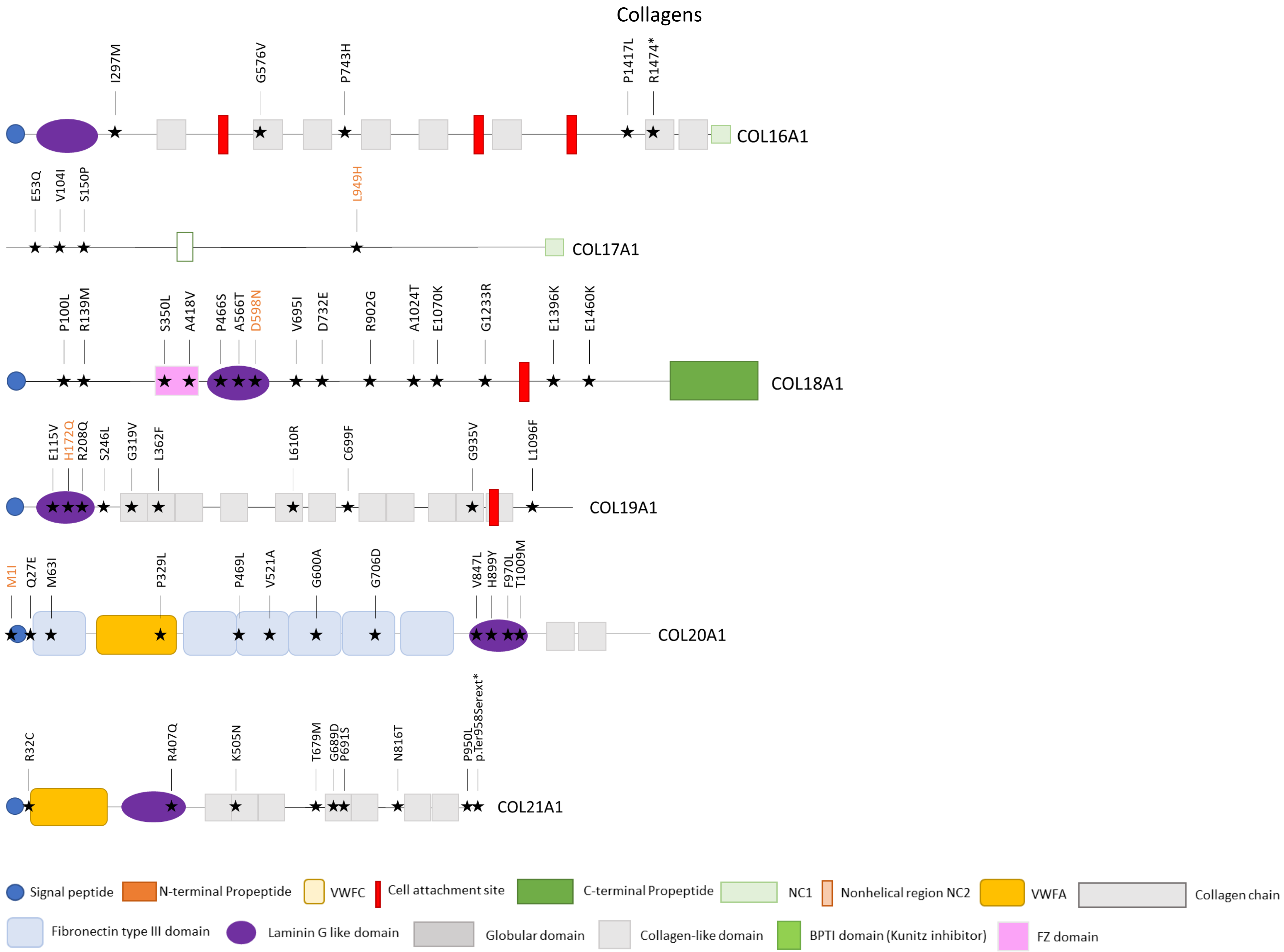

Collagens
I297M
G576V
P743H
P1417L
R1474*
COL16A1
E53Q
V104I
S150P
L949H
COL17A1
P100L
R139M
S350L
A418V
P466S
A566T
D598N
V695I
D732E
R902G
A1024T
E1070K
G1233R
E1396K
E1460K
COL18A1
E115V
H172Q
R208Q
S246L
G319V
L362F
L610R
C699F
G935V
L1096F
COL19A1
M1I
Q27E
M63I
P329L
P469L
V521A
G600A
G706D
V847L
H899Y
F970L
T1009M
COL20A1
R32C
R407Q
K505N
T679M
G689D
P691S
N816T
P950L
p.Ter958Serext*
COL21A1
Signal peptide
N-terminal Propeptide
VWFC
Cell attachment site
C-terminal Propeptide
NC1
Nonhelical region NC2
VWFA
Collagen chain
Fibronectin type III domain
Laminin G like domain
Globular domain
Collagen-like domain
BPTI domain (Kunitz inhibitor)
FZ domain

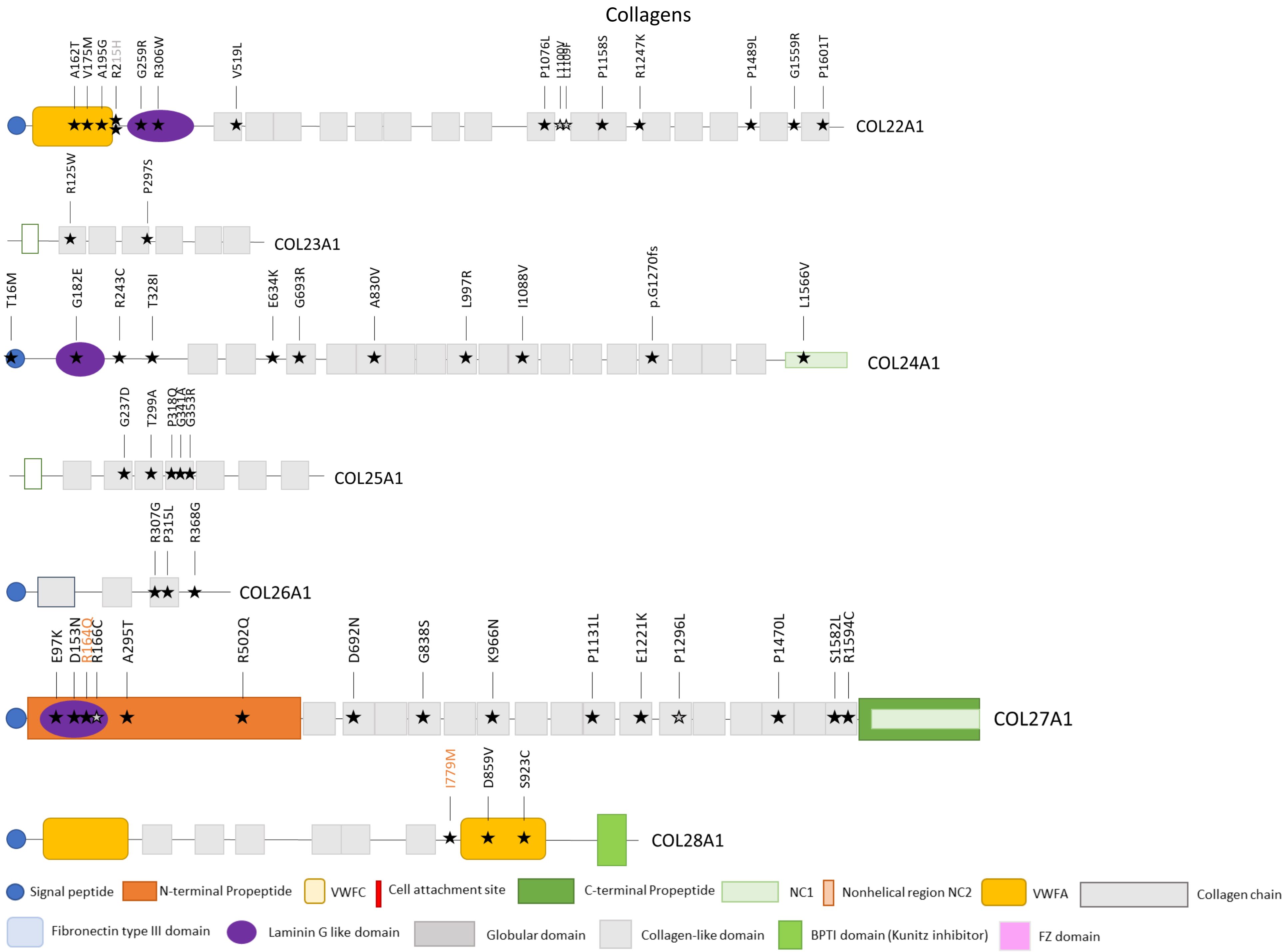

Collagens
A162T
V175M
A195G
R215H
G259R
R306W
V519L
P1076L
P1158S
R1247K
P1489L
G1559R
P1601T
COL22A1
R125W
P297S
COL23A1
T16M
G182E
R243C
T328I
E634K
G693R
A830V
L997R
I1088V
p.G1270fs
L1566V
COL24A1
G237D
T299A
P318Q
G341A
G353R
COL25A1
R307G
P315L
R368G
COL26A1
E97K
D153N
R164Q
R166C
A295T
R502Q
D692N
G838S
K966N
P1131L
E1221K
P1296L
P1470L
S1582L
R1594C
COL27A1
I779M
D859V
S923C
COL28A1
Signal peptide
N-terminal Propeptide
VWFC
Cell attachment site
C-terminal Propeptide
NC1
Nonhelical region NC2
VWFA
Collagen chain
Fibronectin type III domain
Laminin G like domain
Globular domain
Collagen-like domain
BPTI domain (Kunitz inhibitor)
FZ domain

Laminins

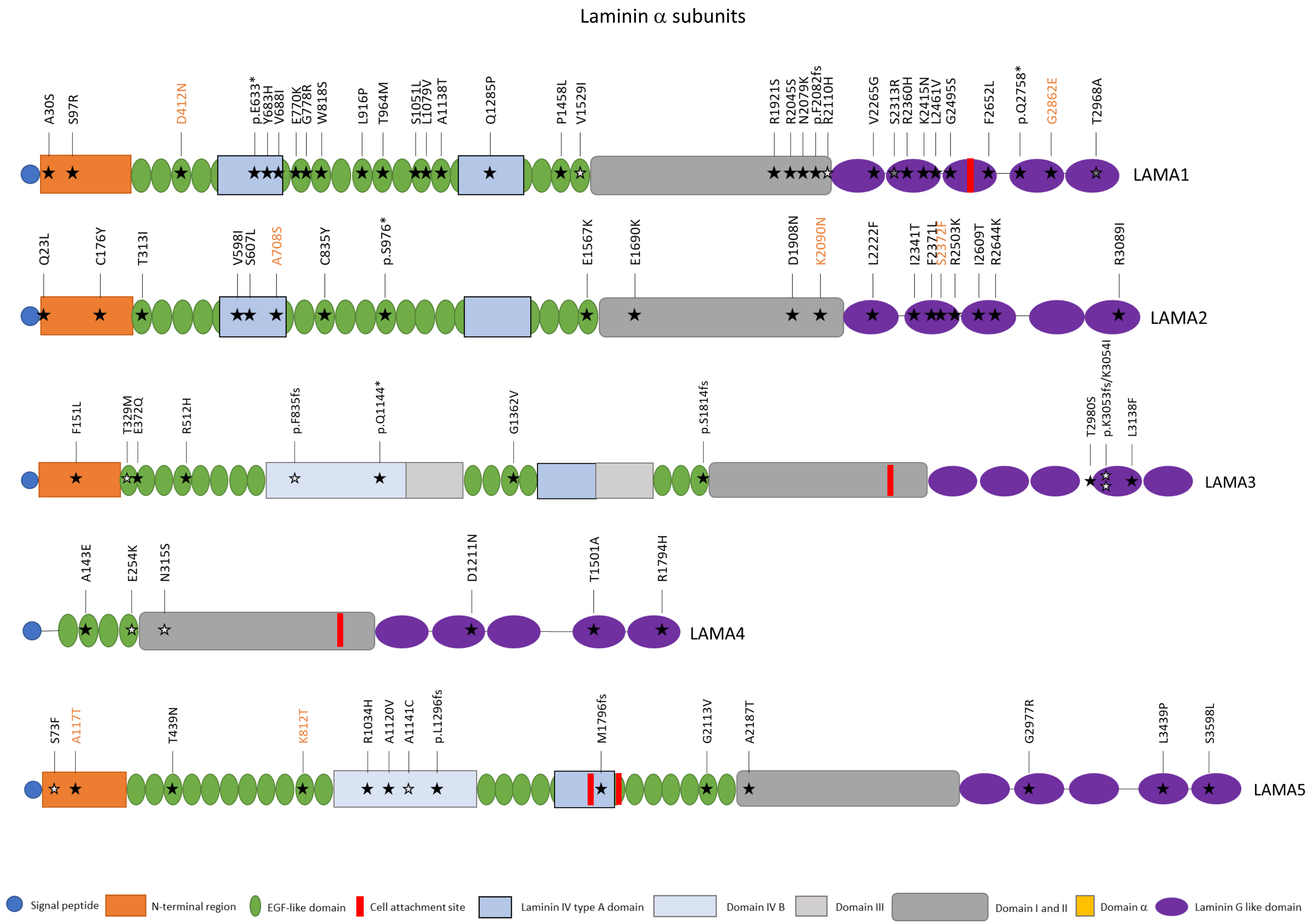

Laminin α subunits
LAMA1
A30S
S97R
D412N
p.E633*
Y683H
V688I
E770K
G778R
W818S
L916P
T964M
S1051L
L1079V
A1138T
Q1285P
P1458L
V1529I
R1921S
R2045S
N2079K
p.F2082fs
R2110H
V2265G
S2313R
R2360H
K2415N
L2461V
G2495S
F2652L
p.Q2758*
G2862E
T2968A
LAMA2
Q23L
C176Y
T313I
V598I
S607L
A708S
C835Y
p.S976*
E1567K
E1690K
D1908N
K2090N
L2222F
I2341T
F2371L
S2372F
R2503K
I2609T
R2644K
R3089I
LAMA3
F151L
T329M
E372Q
R512H
p.F835fs
p.Q1144*
G1362V
p.S1814fs
T2980S
p.K3053fs/K3054I
L3138F
LAMA4
A143E
E254K
N315S
D1211N
T1501A
R1794H
LAMA5
S73F
A117T
T439N
K812T
R1034H
A1120V
A1141C
p.L1296fs
M1796fs
G2113V
A2187T
G2977R
L3439P
S3598L
Signal peptide
N-terminal region
EGF-like domain
Cell attachment site
Laminin IV type A domain
Domain IV B
Domain III
Domain I and II
Domain α
Laminin G like domain

Laminin β subunits

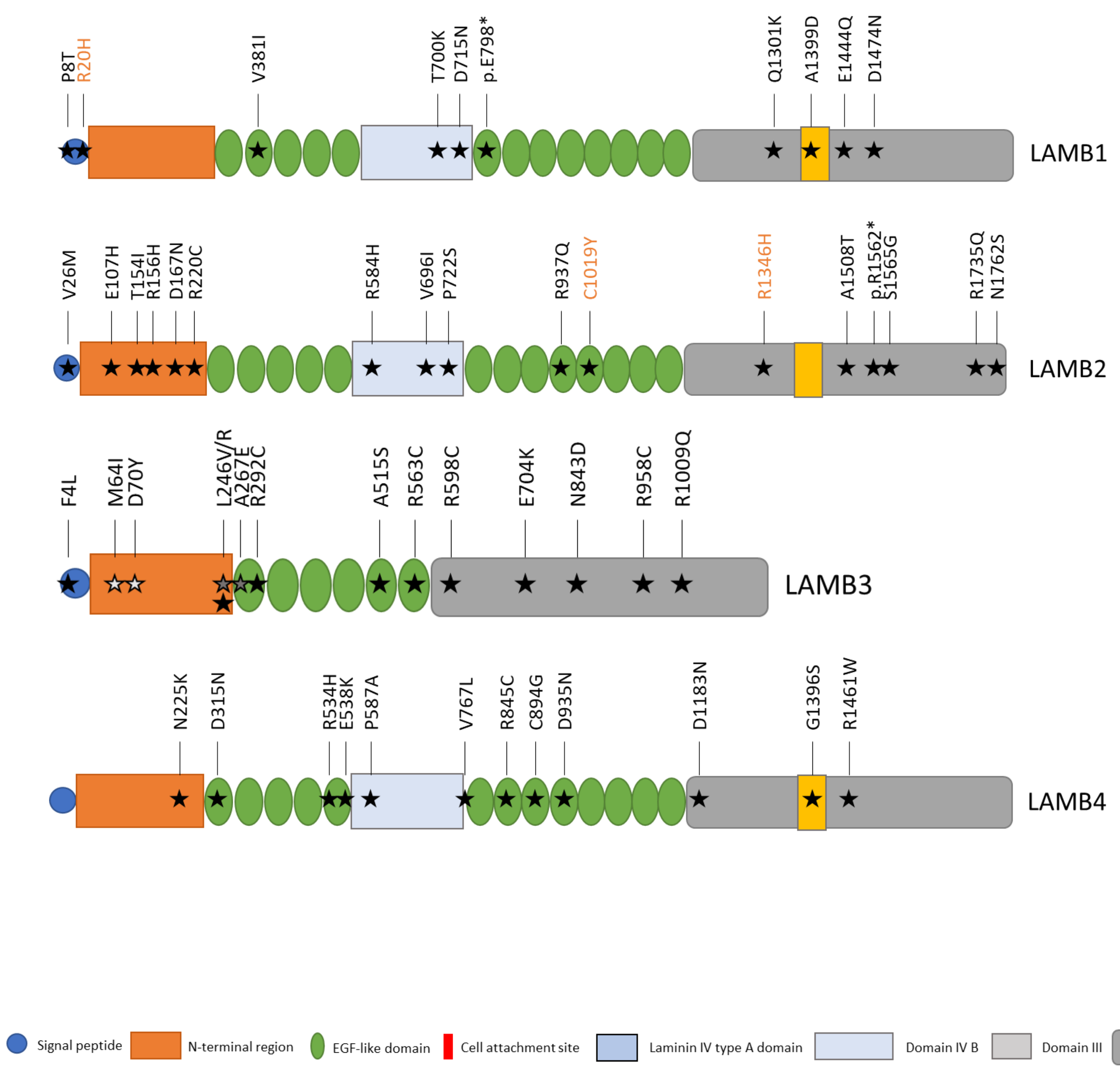

Laminin γ subunits

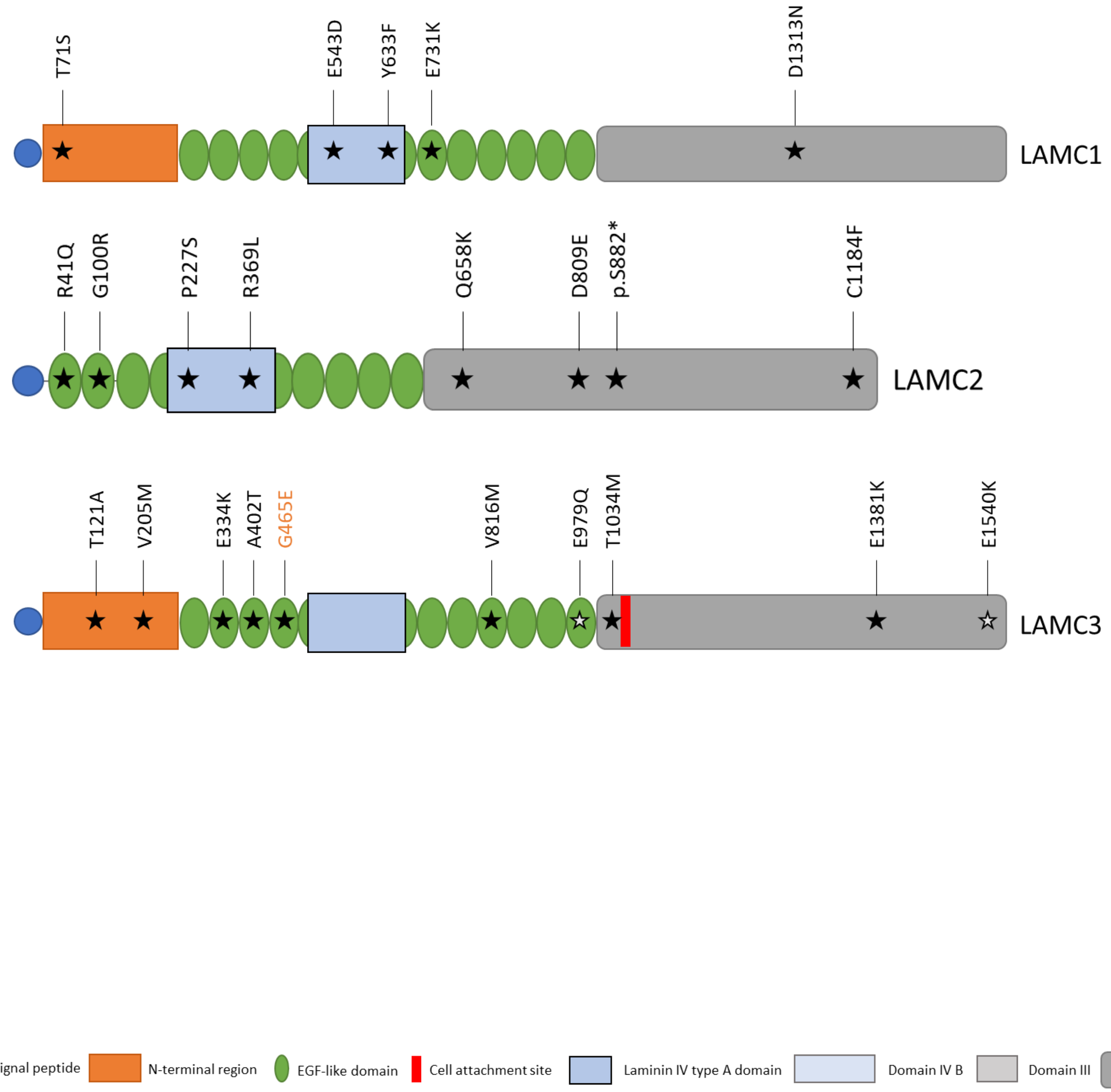

ADAMs

ADAMs

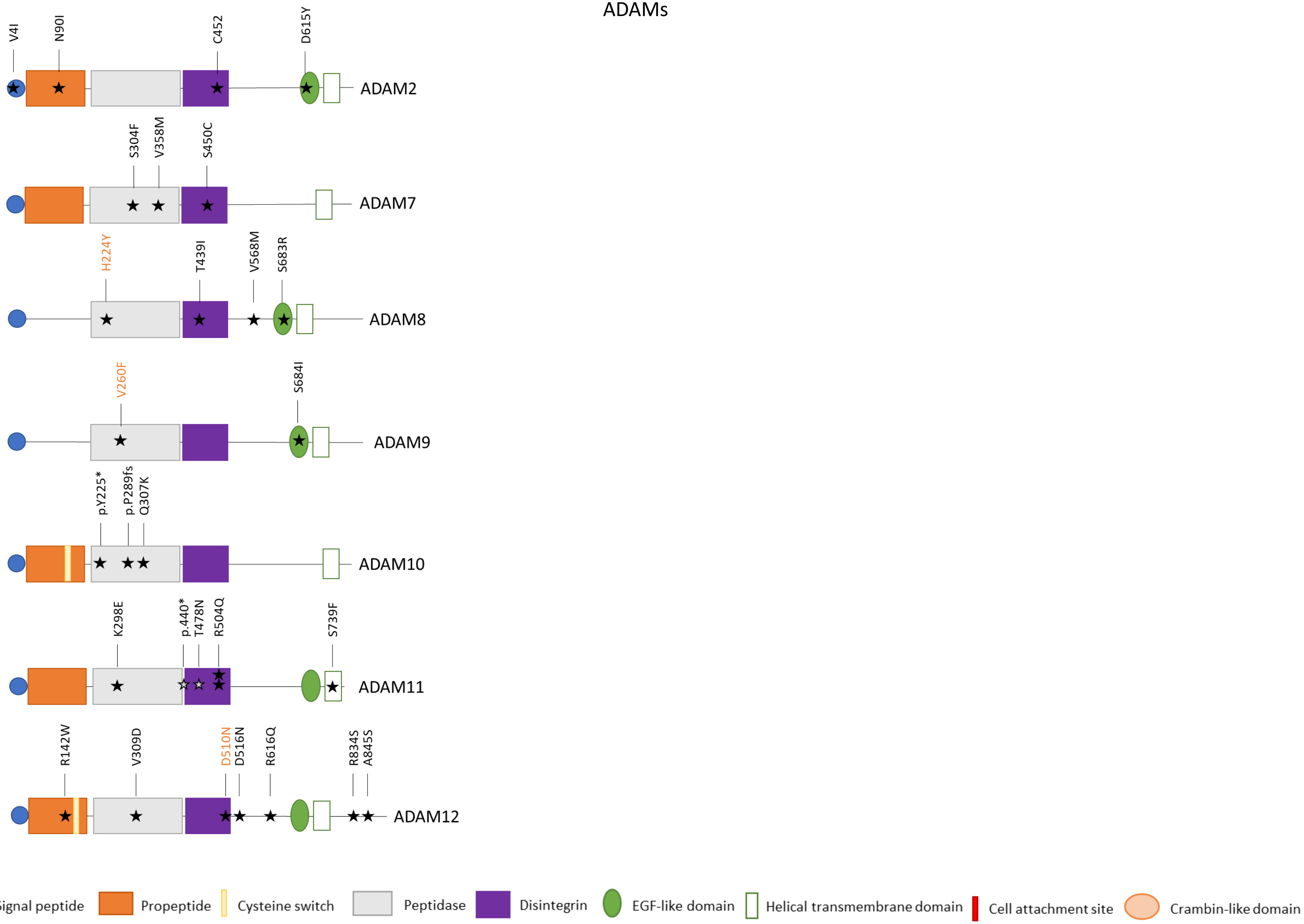

V4I
N90I
C452
D615Y
ADAM2
S304F
V358M
S450C
ADAM7
H224Y
T439I
V568M
S683R
ADAM8
V260F
S684I
ADAM9
p.Y225*
p.P289fs
Q307K
ADAM10
K298E
p.440*
T478N
R504Q
S739F
ADAM11
R142W
V309D
D510N
D516N
R616Q
R834S
A845S
ADAM12
Signal peptide
Propeptide
Cysteine switch
Peptidase
Disintegrin
EGF-like domain
Helical transmembrane domain
Cell attachment site
Crambin-like domain

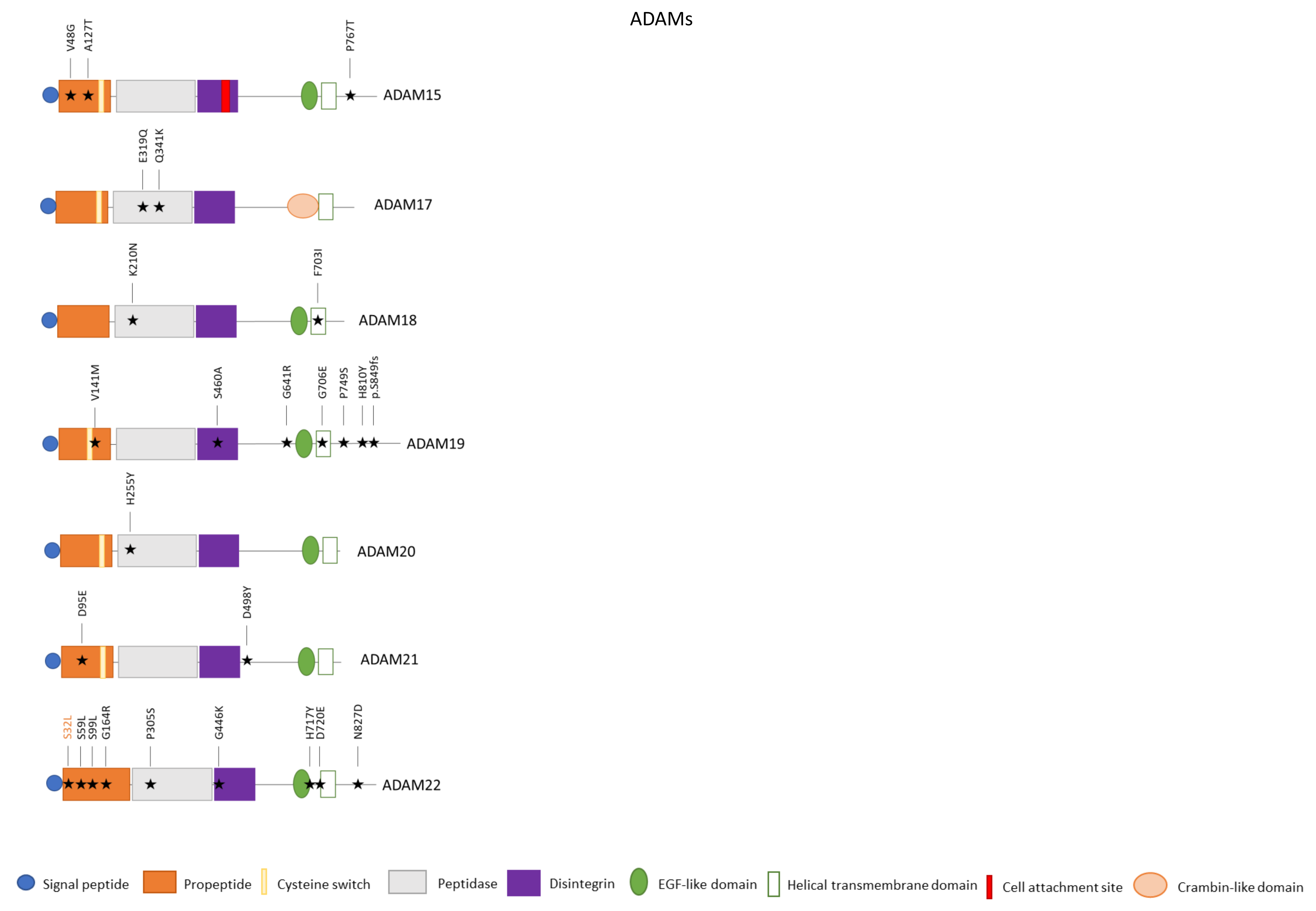
ADAMs
V48G
A127T
P767T
ADAM15
E319Q
Q341K
ADAM17
K210N
F703I
ADAM18
V141M
S460A
G641R
G706E
P749S
H810Y
p.S849fs
ADAM19
H255Y
ADAM20
D95E
D498Y
ADAM21
S32L
S59L
S99L
G164R
P305S
G446K
H717Y
D720E
N827D
ADAM22
Signal peptide
Propeptide
Cysteine switch
Peptidase
Disintegrin
EGF-like domain
Helical transmembrane domain
Cell attachment site
Crambin-like domain

ADAMs

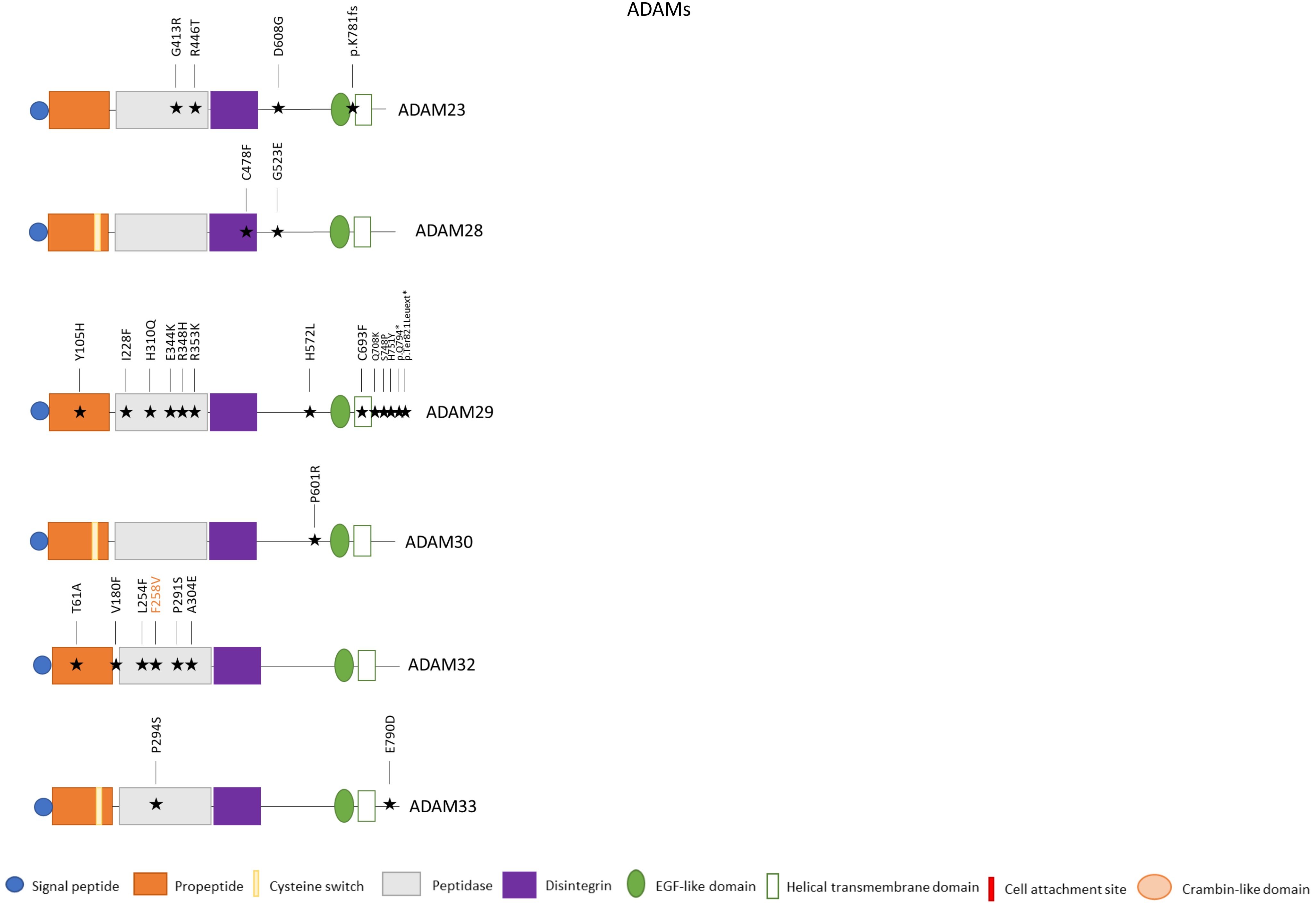
G413R
R446T
D608G
p.K781fs
ADAM23
C478F
G523E
ADAM28
Y105H
I228F
H310Q
E344K
R348H
R353K
H572L
C693F
Q708K
S748P
H751Y
p.Q794*
p.Ter821Leuext*
ADAM29
P601R
ADAM30
T61A
V180F
L254F
F258V
P291S
A304E
ADAM32
P294S
E790D
ADAM33
Signal peptide
Propeptide
Cysteine switch
Peptidase
Disintegrin
EGF-like domain
Helical transmembrane domain
Cell attachment site
Crambin-like domain

ADAMTS

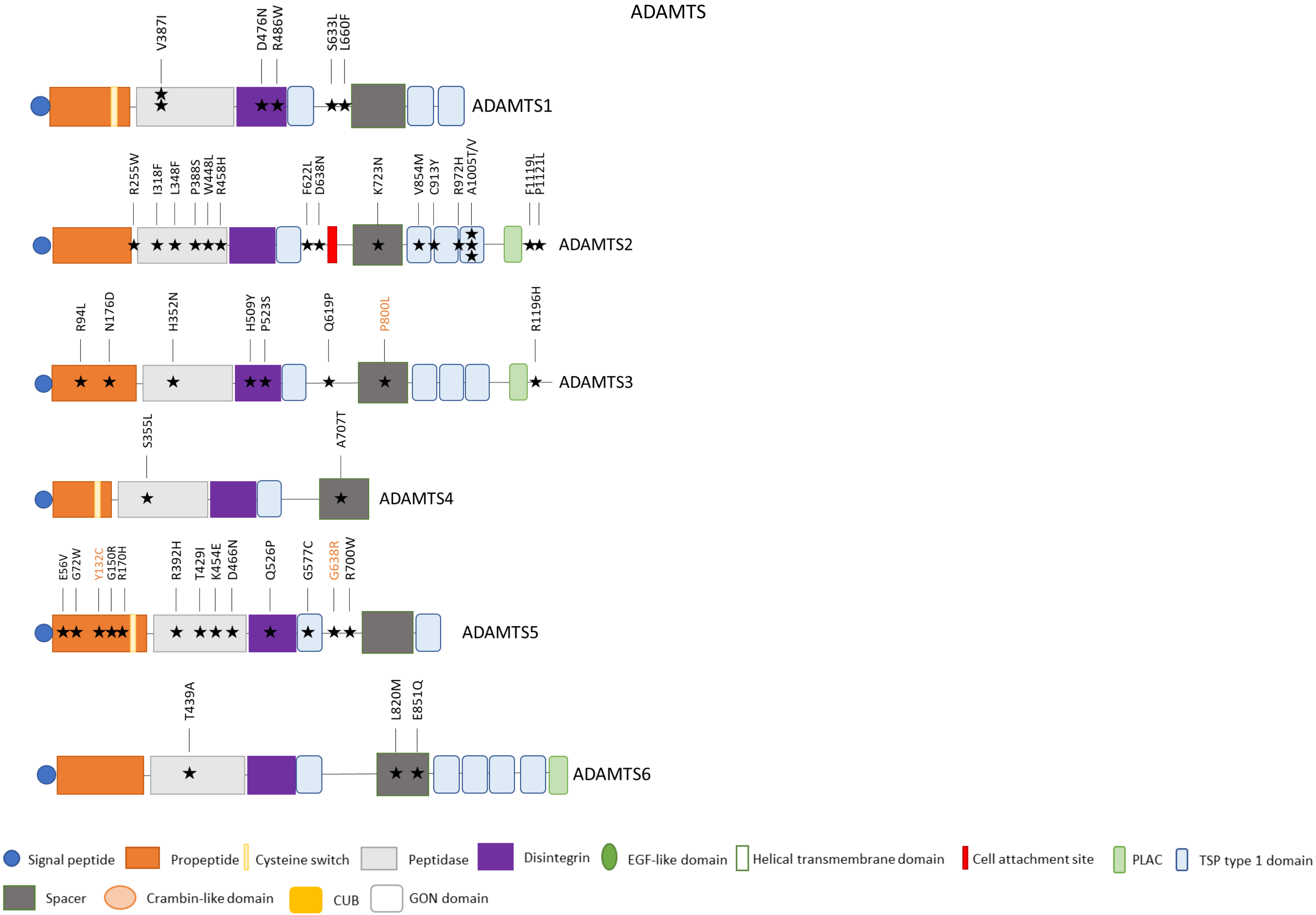
ADAMTS
V387I
D476N
R486W
S633L
L660F
ADAMTS1
R255W
I318F
L348F
P388S
W448L
R458H
F622L
D638N
K723N
V854M
C913Y
R972H
A1005T/V
F1119L
P1121L
ADAMTS2
R94L
N176D
H352N
H509Y
P523S
Q619P
P800L
R1196H
ADAMTS3
S355L
A707T
ADAMTS4
E56V
G72W
Y132C
G150R
R170H
R392H
T429I
K454E
D466N
Q526P
G577C
G638R
R700W
ADAMTS5
T439A
L820M
E851Q
ADAMTS6
Signal peptide
Propeptide
Cysteine switch
Peptidase
Disintegrin
EGF-like domain
Helical transmembrane domain
Cell attachment site
PLAC
TSP type 1 domain
Spacer
Crambin-like domain
CUB
GON domain

ADAMTS

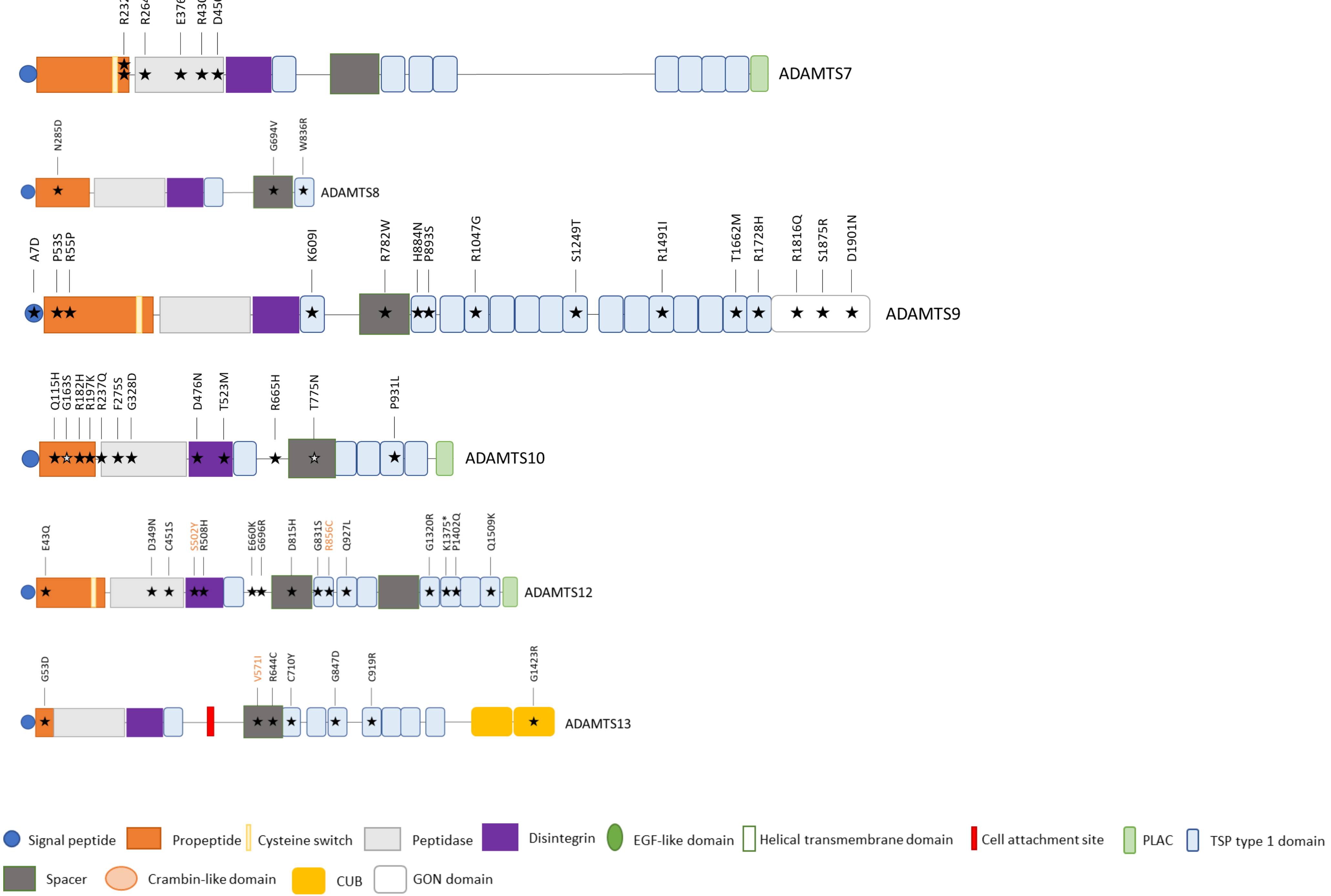

R232H
R264K
E376Q
R430C
D450N
ADAMTS7
N285D
G694V
W836R
ADAMTS8
A7D
P53S
R55P
K609I
R782W
H884N
P893S
R1047G
S1249T
R1491I
T1662M
R1728H
R1816Q
S1875R
D1901N
ADAMTS9
Q115H
G163S
R182H
R197K
R237Q
F275S
G328D
D476N
T523M
R665H
T775N
P931L
ADAMTS10
E43Q
D349N
C451S
S502Y
R508H
E660K
G696R
D815H
G831S
R856C
Q927L
G1320R
K1375*
P1402Q
Q1509K
ADAMTS12
G53D
V571I
R644C
C710Y
G847D
C919R
G1423R
ADAMTS13
Signal peptide
Propeptide
Cysteine switch
Peptidase
Disintegrin
EGF-like domain
Helical transmembrane domain
Cell attachment site
PLAC
TSP type 1 domain
Spacer
Crambin-like domain
CUB
GON domain

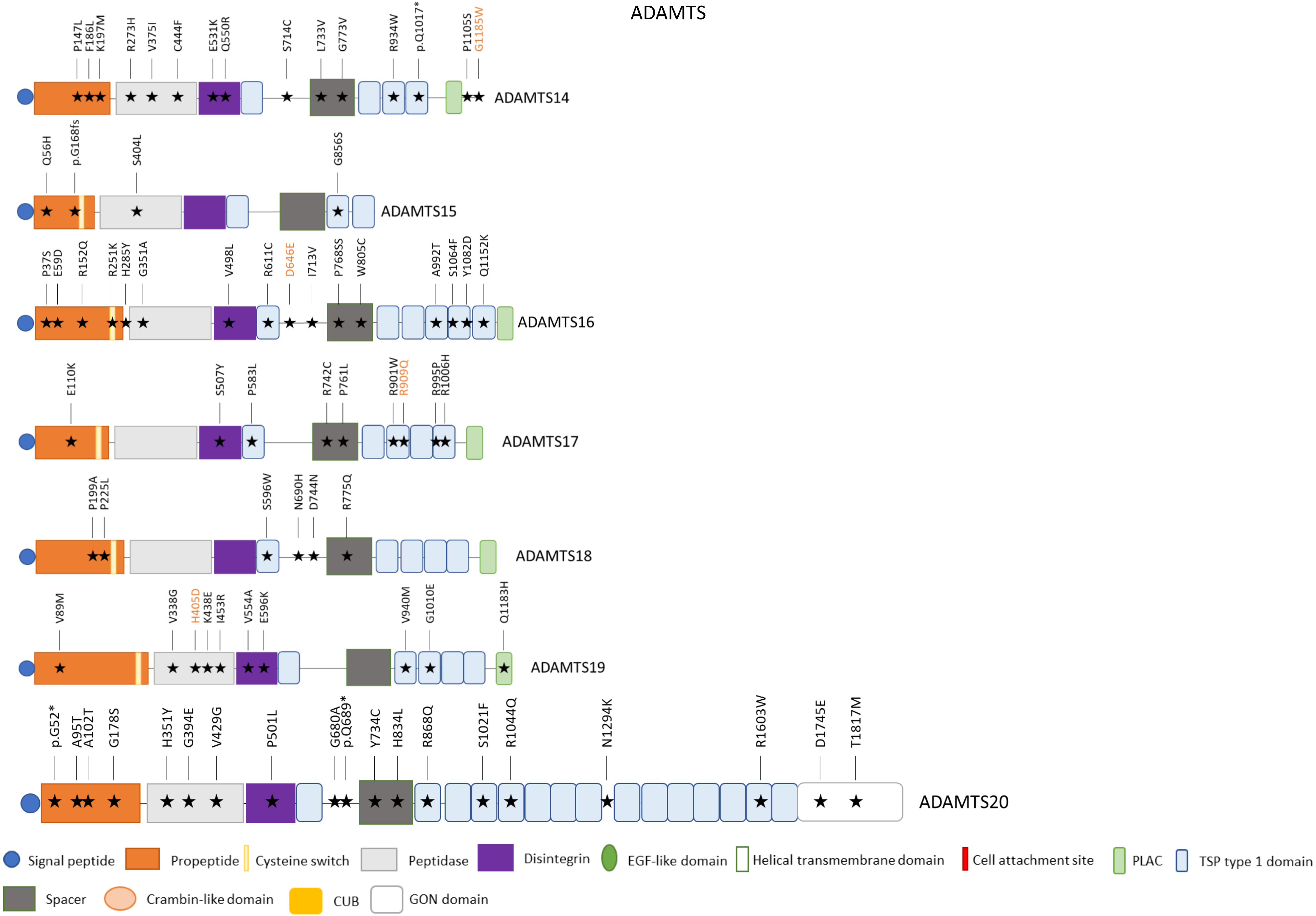
ADAMTS
P147L
F186L
K197M
R273H
V375I
C444F
E531K
Q550R
S714C
L733V
G773V
R934W
p.Q1017*
P1105S
G1185W
ADAMTS14
Q56H
p.G168fs
S404L
G856S
ADAMTS15
P37S
E59D
R152Q
R251K
H285Y
G351A
V498L
R611C
D646E
I713V
P768SS
W805C
A992T
S1064F
Y1082D
Q1152K
ADAMTS16
E110K
S507Y
P583L
R742C
P761L
R901W
R909Q
R995P
R1006H
ADAMTS17
P199A
P225L
S596W
N690H
D744N
R775Q
ADAMTS18
V89M
V338G
H405D
K438E
I453R
V554A
E596K
V940M
G1010E
Q1183H
ADAMTS19
p.G52*
A95T
A102T
G178S
H351Y
G394E
V429G
P501L
G680A
p.Q689*
Y734C
H834L
R868Q
S1021F
R1044Q
N1294K
R1603W
D1745E
T1817M
ADAMTS20
Signal peptide
Propeptide
Cysteine switch
Peptidase
Disintegrin
EGF-like domain
Helical transmembrane domain
Cell attachment site
PLAC
TSP type 1 domain
Spacer
Crambin-like domain
CUB
GON domain

Figure S2: Kaplan Meier plots for the comparison of overall and progression-free survival of MMRF patients with a gene expression (GE) higher or lower than the mean/median expression measured across all samples. Statistical correlations were performed using the Log Rank test. *P* values were adjusted for multiple hypothesis testing using Benjamini-Hochberg correction in R. n=800. Graphs were created using GraphPad Prism 9.

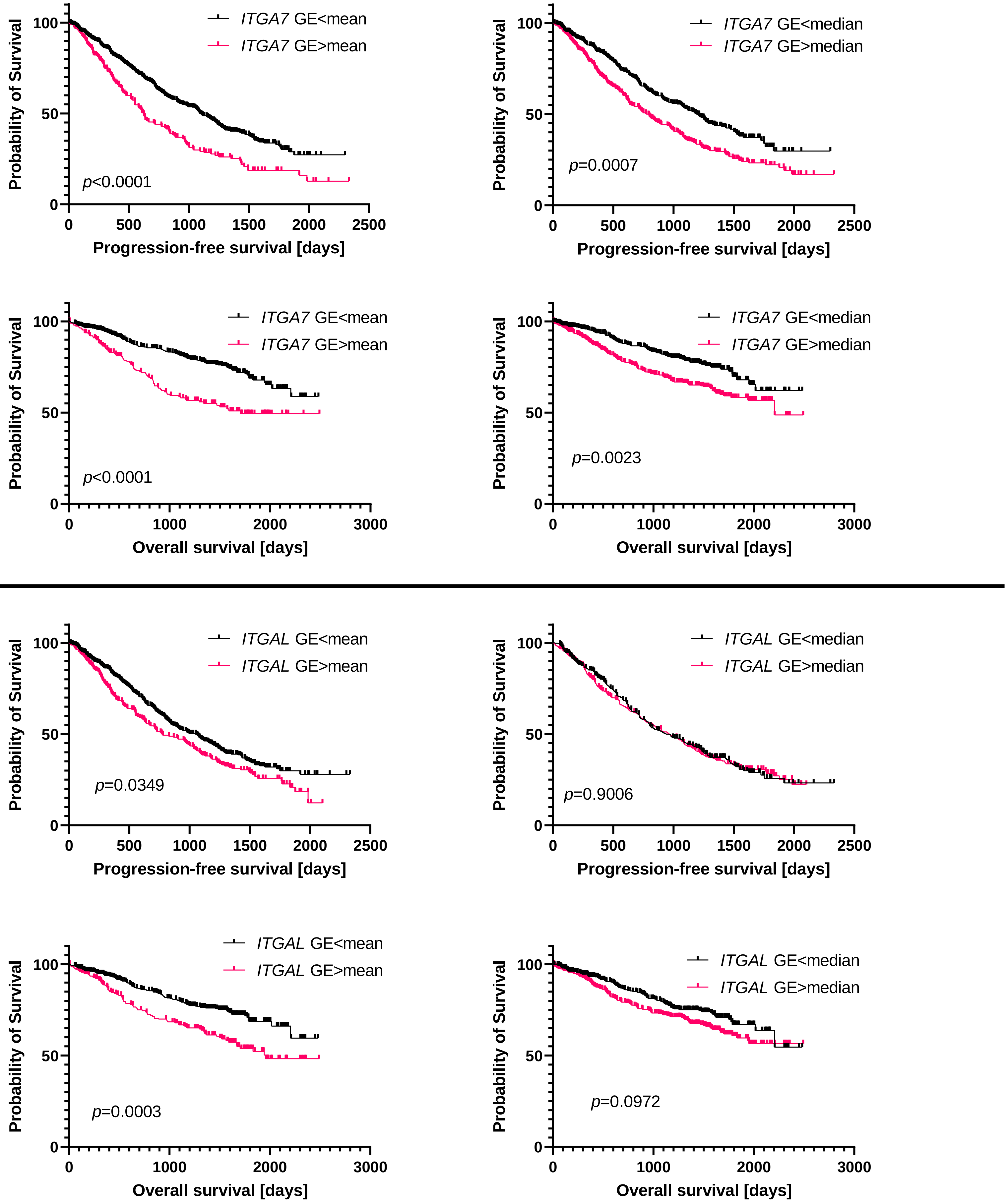

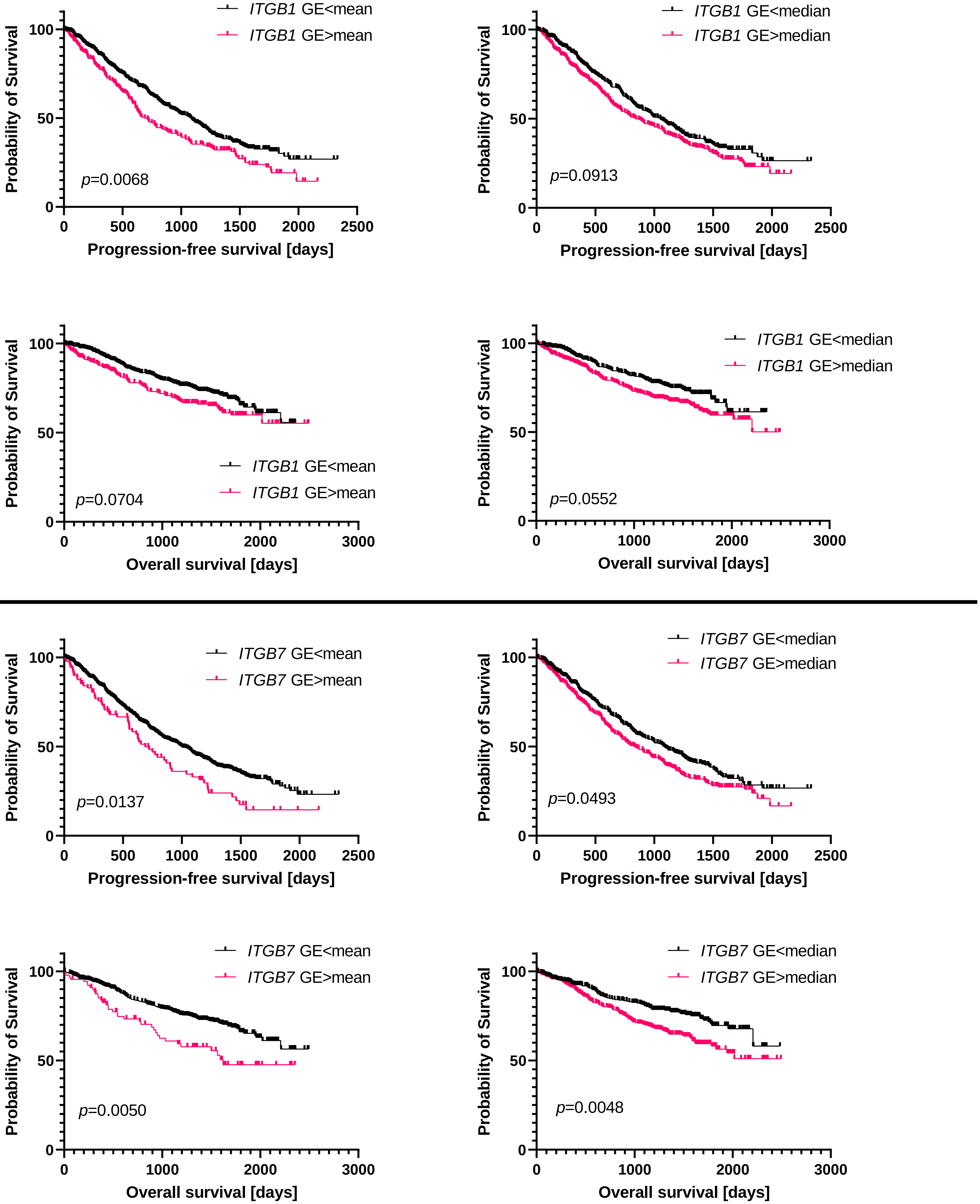
ITGB1 GE<mean
ITGB1 GE>mean
p=0.0068
Probability of Survival
Progression-free survival [days]
ITGB1 GE<median
ITGB1 GE>median
p=0.0913
Probability of Survival
Progression-free survival [days]
ITGB1 GE<mean
ITGB1 GE>mean
p=0.0704
Probability of Survival
Overall survival [days]
ITGB1 GE<median
ITGB1 GE>median
p=0.0552
Probability of Survival
Overall survival [days]
ITGB7 GE<mean
ITGB7 GE>mean
p=0.0137
Probability of Survival
Progression-free survival [days]
ITGB7 GE<median
ITGB7 GE>median
p=0.0493
Probability of Survival
Progression-free survival [days]
ITGB7 GE<mean
ITGB7 GE>mean
p=0.0050
Probability of Survival
Overall survival [days]
ITGB7 GE<median
ITGB7 GE>median
p=0.0048
Probability of Survival
Overall survival [days]

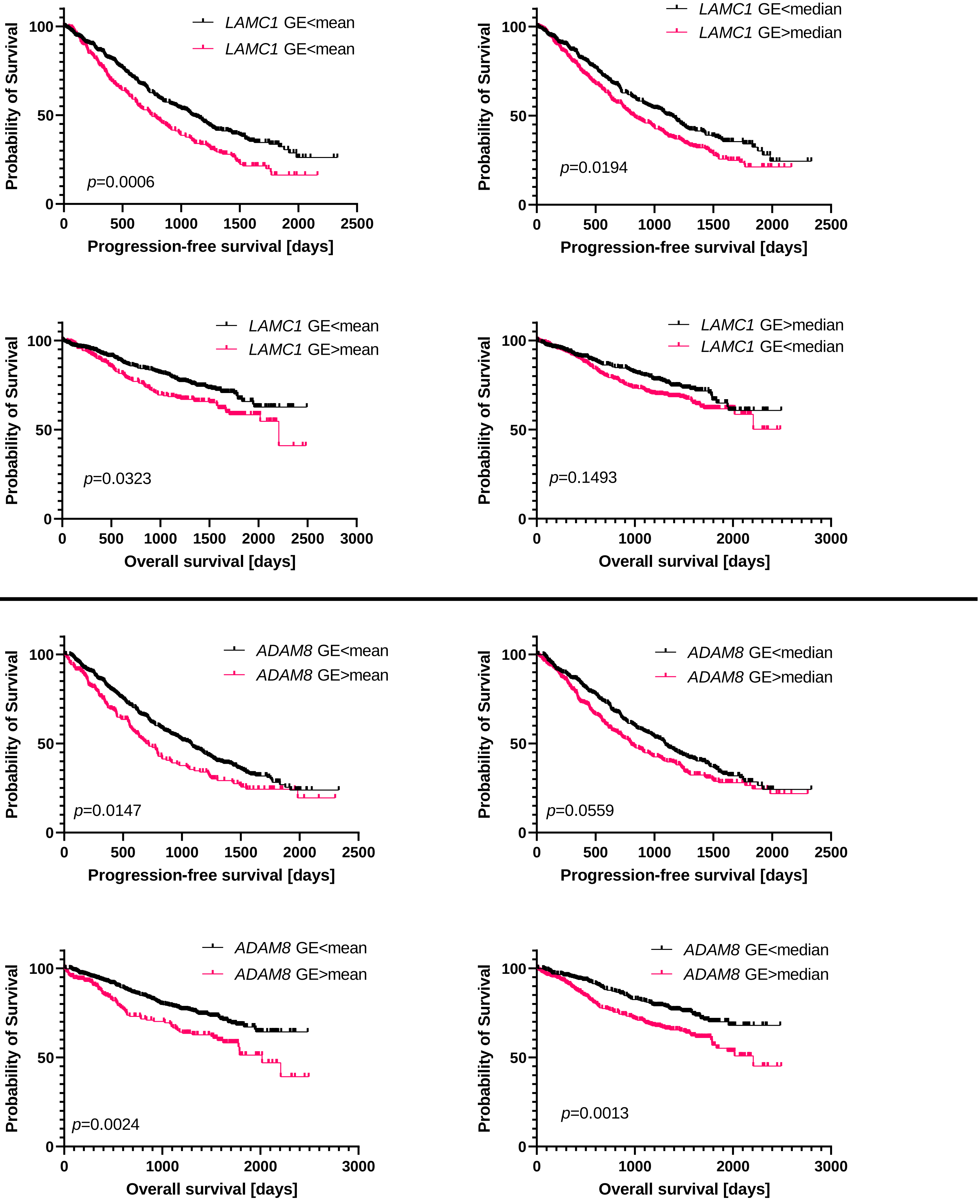
Probability of Survival
100
50
0
LAMC1 GE<mean
LAMC1 GE<mean
p=0.0006
0 500 1000 1500 2000 2500
Progression-free survival [days]
Probability of Survival
100
50
0
LAMC1 GE<median
LAMC1 GE>median
p=0.0194
0 500 1000 1500 2000 2500
Progression-free survival [days]
Probability of Survival
100
50
0
LAMC1 GE<mean
LAMC1 GE>mean
p=0.0323
0 500 1000 1500 2000 2500 3000
Overall survival [days]
Probability of Survival
100
50
0
LAMC1 GE>median
LAMC1 GE<median
p=0.1493
0 1000 2000 3000
Overall survival [days]
Probability of Survival
100
50
0
ADAM8 GE<mean
ADAM8 GE>mean
p=0.0147
0 500 1000 1500 2000 2500
Progression-free survival [days]
Probability of Survival
100
50
0
ADAM8 GE<median
ADAM8 GE>median
p=0.0559
0 500 1000 1500 2000 2500
Progression-free survival [days]
Probability of Survival
100
50
0
ADAM8 GE<mean
ADAM8 GE>mean
p=0.0024
0 1000 2000 3000
Overall survival [days]
Probability of Survival
100
50
0
ADAM8 GE<median
ADAM8 GE>median
p=0.0013
0 1000 2000 3000
Overall survival [days]

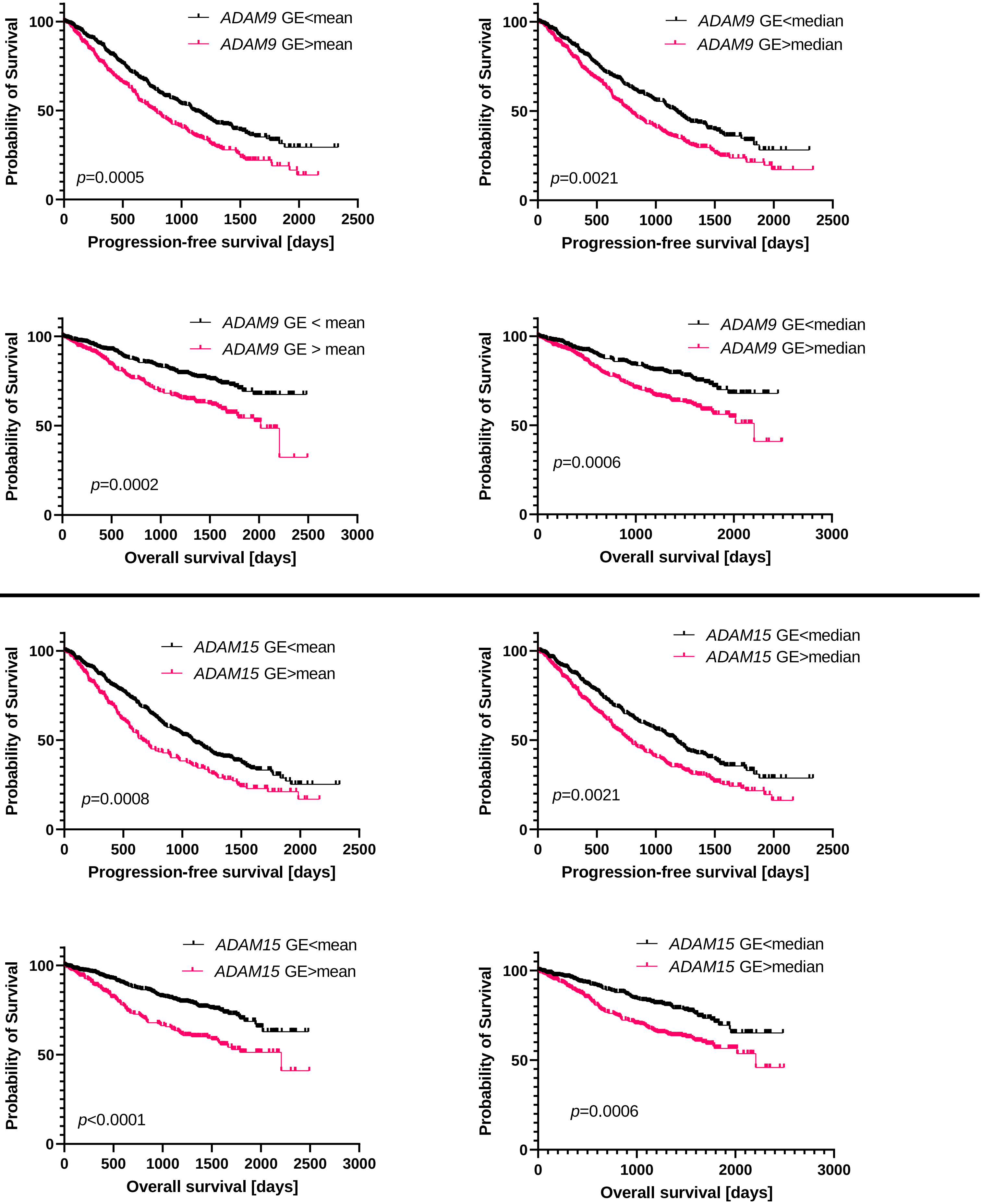
ADAM9 GE<mean
ADAM9 GE>mean
p=0.0005
Probability of Survival
Progression-free survival [days]
ADAM9 GE<median
ADAM9 GE>median
p=0.0021
Probability of Survival
Progression-free survival [days]
ADAM9 GE < mean
ADAM9 GE > mean
p=0.0002
Probability of Survival
Overall survival [days]
ADAM9 GE<median
ADAM9 GE>median
p=0.0006
Probability of Survival
Overall survival [days]
ADAM15 GE<mean
ADAM15 GE>mean
p=0.0008
Probability of Survival
Progression-free survival [days]
ADAM15 GE<median
ADAM15 GE>median
p=0.0021
Probability of Survival
Progression-free survival [days]
ADAM15 GE<mean
ADAM15 GE>mean
p<0.0001
Probability of Survival
Overall survival [days]
ADAM15 GE<median
ADAM15 GE>median
p=0.0006
Probability of Survival
Overall survival [days]

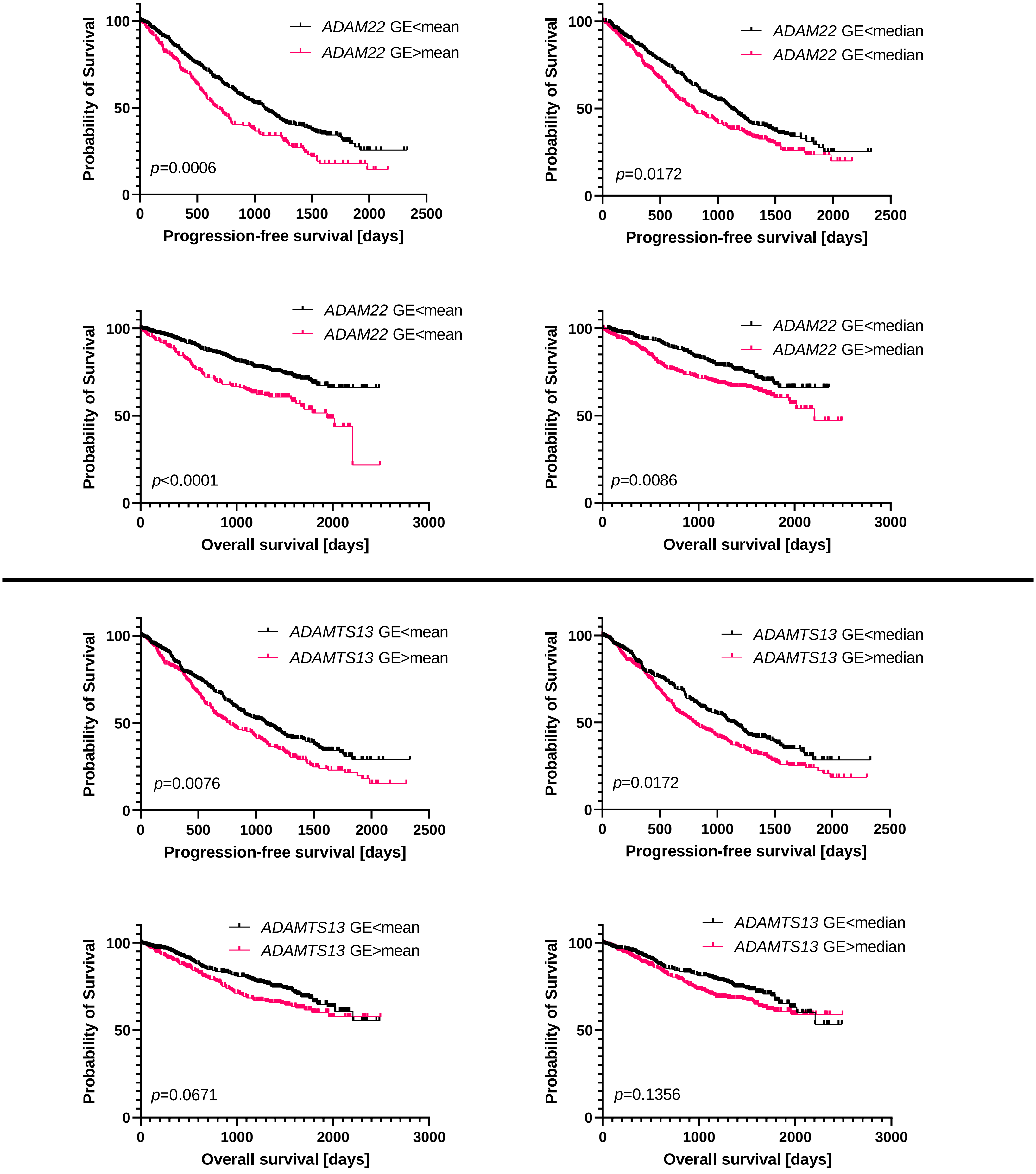

Probability of Survival
ADAM22 GE<mean
ADAM22 GE>mean
p=0.0006
Progression-free survival [days]
ADAM22 GE<median
ADAM22 GE<median
p=0.0172
Progression-free survival [days]
ADAM22 GE<mean
ADAM22 GE<mean
p<0.0001
Overall survival [days]
ADAM22 GE<median
ADAM22 GE>median
p=0.0086
Overall survival [days]
ADAMTS13 GE<mean
ADAMTS13 GE>mean
p=0.0076
Progression-free survival [days]
ADAMTS13 GE<median
ADAMTS13 GE>median
p=0.0172
Progression-free survival [days]
ADAMTS13 GE<mean
ADAMTS13 GE>mean
p=0.0671
Overall survival [days]
ADAMTS13 GE<median
ADAMTS13 GE>median
p=0.1356
Overall survival [days]
